# A systematic review of the biological, social, and environmental determinants of intellectual disability in children and adolescents

**DOI:** 10.3389/fpsyt.2022.926681

**Published:** 2022-08-25

**Authors:** Helen Leonard, Alicia Montgomery, Brittany Wolff, Elissa Strumpher, Anne Masi, Susan Woolfenden, Katrina Williams, Valsamma Eapen, Amy Finlay-Jones, Andrew Whitehouse, Martyn Symons, Melissa Licari, Kandice Varcin, Gail Alvares, Kiah Evans, Jenny Downs, Emma Glasson

**Affiliations:** ^1^Telethon Kids Institute, Centre for Child Health Research, The University of Western Australia, Perth, WA, Australia; ^2^School of Clinical Medicine, University of New South Wales, Sydney, NSW, Australia; ^3^Department of Paediatrics, Monash University, Clayton, VIC, Australia

**Keywords:** intellectual disability, systematic review, sociodemographic, antenatal, risk factor, maternal health, data linkage, population data

## Abstract

**Aim:**

This systematic review aimed to identify the most important social, environmental, biological, and/or genetic risk factors for intellectual disability (ID).

**Methods:**

Eligible were published prospective or retrospective comparative studies investigating risk factors for ID in children 4–18 years. Exclusions were single group studies with no comparator without ID and a sample size <100. Electronic databases (Medline, Cochrane Library, EMBASE, PsycInfo, Campbell Collaboration, and CINAHL) were searched for eligible publications from 1980 to 2020. Joanna Briggs Institute critical appraisal instruments, appropriate for study type, were used to assess study quality and risk of bias. Descriptive characteristics and individual study results were presented followed by the synthesis for individual risk factors, also assessed using GRADE.

**Results:**

Fifty-eight individual eligible studies were grouped into six exposure topics: sociodemographic; antenatal and perinatal; maternal physical health; maternal mental health; environmental; genetic or biological studies. There were few eligible genetic studies. For half the topics, the certainty of evidence (GRADE) was moderate or high.

**Conclusion:**

Multiple studies have examined individual potential determinants of ID, but few have investigated holistically to identify those populations most at risk. Our review would indicate that there are vulnerable groups where risk factors we identified, such as low socioeconomic status, minority ethnicity, teenage motherhood, maternal mental illness, and alcohol abuse, may cluster, highlighting a target for preventive strategies. At-risk populations need to be identified and monitored so that interventions can be implemented when appropriate, at preconception, during pregnancy, or after birth. This could reduce the likelihood of ID and provide optimal opportunities for vulnerable infants.

**Systematic review registration:**

[https://www.crd.york.ac.uk/prospero/display_record.php?RecordID=120032], identifier [CRD42019120032].

## Introduction

Intellectual disability (ID) is a neurodevelopmental disorder (NDD) characterized by impairments in cognitive function and adaptive functioning, manifest prior to the age of 18 years (1). The diagnosis of ID is generally made on the basis of intelligence quotient (IQ) scores, derived from standardized measures of intelligence (2). ID is defined as approximately two standard deviations or more below the population mean, which is equivalent to an IQ score of approximately 70 or less (1). Diagnosis also requires associated impairment in adaptive functioning (approximately two standard deviations or more below the population mean on standardized measures) and onset in childhood (2). The classification of severity of ID is often defined as mild, moderate, severe, or profound on the basis of IQ scores and the intensity of associated support needs (3). More recently, greater emphasis has been placed on the impact of ID on adaptive function (4). Accordingly, the Diagnostic and Statistical Manual for Mental Disorders—5th Edition (DSM-5), defines ID severity on the basis of adaptive functioning, rather than IQ scores alone (5).

The prevalence of ID varies between studies, geographical location, and time periods, impacted by different criteria and cutoff scores used to define ID (4). A recent meta-analysis across low-, middle-, and high-income countries reported an overall prevalence of 18.3/1,000 (6). One of the few population-based studies worldwide found that the prevalence of ID has risen over the last decade, from 14.3/1,000 between 1983 and 1992 (7) to 17.0/1,000 livebirths between 1983 and 2005 (2). This increase may be explained, at least in part, by the increase in the prevalence of autism spectrum disorder (ASD) over the same period, as up to 70% of individuals with a diagnosis of ASD had comorbid ID (2). The individual, family, and societal burden associated with ID is immense, with high associated health service needs (8–11).

The etiology of ID is poorly understood, and both genetic and environmental factors are implicated. In some cases, it is attributed to identifiable disorders, such as a genetic anomaly, for example, Down syndrome, or *in utero* exposure to infection or teratogens, such as alcohol. However, in 50–60% of cases (or more for mild ID), no associated genetic or medical disorder is identified (2, 7, 12–14). Individuals with ID also commonly present with additional neurodevelopmental problems and may also be diagnosed with ASD or cerebral palsy (CP) (15). Additional health conditions, such as epilepsy, often co-occur resulting in considerable heterogeneity in presentation.

Myriad social, biological, and genetic risk factors have been associated with ID. Demographic factors associated with an increased likelihood of ID include male sex (16), lower socioeconomic status (6, 17, 18), and ethnic minorities (2). Numerous pre- and perinatal factors have also been implicated, including advanced maternal age, high parity, maternal alcohol use, maternal tobacco use, gestational diabetes, maternal hypertension, preterm birth, and low birthweight (19). Other biological risks relate to factors, such as exposure to teratogens, viruses, or trauma (20). Genetic risk factors include a broad range of chromosomal abnormalities, autosomal trisomies, aneuploidies of the X-chromosome, and pathogenic copy number variants (14, 20).

At least some of the identifiable risk factors may be on the causal pathway for ID, either from biological perspectives, or as social determinants. Identification of known risk factors enables holistic consideration as to which factors may be modifiable. It has been shown that a small proportion of a childhood population can contribute to a large proportion of adult health burden (21). Similarly, it is possible that certain combinations of risk factors could disproportionally contribute to the burden of ID. This knowledge could be used to develop risk indices which can be applied on a population level to identify the most vulnerable sectors of the community, where there will be the most gain from early intervention but where there is the highest risk of non-participation in universal developmental surveillance (22, 23). The concept of proportionate universalism (24) provides a framework where universal services, both antenatally and into infancy, can be supplemented with additional services directed at those at highest risk. Thereby these populations can be supported in efforts to reduce the likelihood of ID or at least to reduce its impact. The purpose of this systematic review was thus to identify the most important social, environmental, biological, and/or genetic factors which will help to characterize these high-risk groups.

## Methods

This systematic review was conducted and reported in accordance with the Preferred Reporting Items for Systematic Reviews and Meta-Analyses (PRISMA) Statement (25, 26). The study protocol was developed *a priori* and registered with PROSPERO (Registration number: CRD 42019120032).

### Search strategy

**FIGURE 1:**
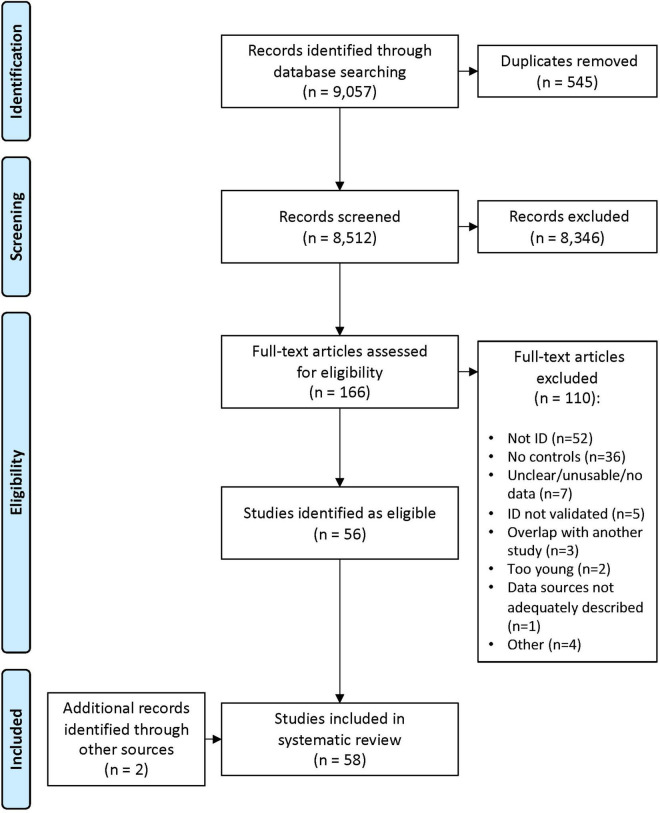
Preferred reporting items for systematic reviews and meta-analyses (PRISMA) flow diagram of the literature review and paper inclusion pathways.

The search range was 1980–2020 (initial search conducted in January 2019; with an extended search conducted in October 2020 of any eligible articles published between January 2019 and October 2020). The databases searched were Medline 1996—present (Ovid platform), Cochrane Library (Wiley platform), EMBASE, PsycInfo, Campbell Collaboration, and the Cumulative Index to Nursing and Allied Health Literature (CINAHL). This review sought to identify the breadth of potential risk factors for ID, including (but not limited to) individual child characteristics (biological/genetic risk factors), family characteristics (maternal age at birth, education, income, socioeconomic status, single-parent status, family size, parental mental health, and social capital/support), socio-cultural factors (membership to culturally and linguistically diverse populations, migrant populations, or indigenous populations), and other societal and geographical factors. Expander terms, such as MeSH headings and explode, were used to broaden the search in each database. The search terms and strategy are documented in Supplementary Table 1. Duplicates were removed, and abstracts were screened (ES, HL). The full texts of remaining articles were then assessed for eligibility against the inclusion and exclusion criteria (ES, HL, and EG). At each stage, for studies where eligibility was unclear, a collaborative decision was reached through discussion. Reasons for exclusion at the full-text stage were recorded. Included studies underwent a process of critical assessment (see quality assessment). The results of the search, including the numbers of articles at each stage and reasons for exclusion at the full-text stage, are presented in a PRISMA flow diagram (Figure 1).

### Eligibility criteria

Published, peer-reviewed reports of prospective or retrospective comparative studies, including birth cohort studies, which examined risk factors for ID in children aged 4–18 years of age were included to ensure a valid IQ score could be obtained. For the purpose of this systematic review, ID was defined as an IQ of 70 or less (approximately two standard deviations below the mean), associated difficulties with adaptive functioning, and the condition manifesting prior to the age of 18 years. Articles that included individuals with ID and any other comorbid condition, including but not limited to autism, fetal alcohol spectrum disorder, cerebral palsy, epilepsy, and known genetic syndromes, were eligible. Articles including children with a diagnosis of developmental delay without ID diagnosis were not eligible. Where comorbid disorders were the focus of an article and individuals both with and without ID were included in the sample, articles were only included if subgroup data were presented, pertaining specifically to the subgroup with ID. The comparison group, or controls, were subjects without ID as defined above. Studies were excluded if they were single group studies with no comparator without ID, the sample size was less than 100, or the article was published before 1980.

The primary outcomes were factors that were identified to be significantly associated with the risk of ID in children and adolescents, as well as the strength of these associations. These determinants relate to individual, familial, biological, genetic, social, or environmental factors.

### Data extraction

Data were extracted regarding the country, study design, data source and time period, sample size (including number with ID), method of assigning ID status and exclusions, comparison group, risk factors examined (and their categorization), covariates, analysis type, and risk factors identified. Data were extracted independently by three reviewers (ES, HL, and EG) using a standardized data extraction form. Any disagreements were discussed until a consensus was reached.

### Quality assessment

All individual studies were critically appraised by two of seven independent assessors (VE, EG, HL, BM, AMa, AMo, and SW) at the study level for methodological quality using standardized critical appraisal instruments from the Joanna Briggs Institute (27). Three tools were used according to study type (cohort, cross-sectional, and case–control), for which each criterion was rated “Yes,” “No,” or “Unsure.” The “Yes” ratings were tallied for each study, and any disagreements were resolved through the input of a third assessor. Quality assessment ratings were standardized across study type by allocating a *low*, *medium*, or *high* level of quality for each study according to the percent of “Yes” ratings received (low < 75%, medium 75–89%, and high > 89%). Furthermore, using methodology similar to previous research (Supplementary Table 2) (28), we used the GRADE approach to assess the quality of evidence for specific outcomes as opposed to specific studies (as was done using the Joanna Briggs tools). We restricted GRADE assessments to those where the risk factors and levels of ID were comparable across studies. Given that for observational studies the maximum baseline level of evidence is considered to be moderate, a high level of certainty is difficult to achieve.

### Data synthesis

We present descriptive characteristics of the individual studies in Table 1, which included type of study, data source, time period, sample size including number with ID, comparator group, criteria for assessing ID, and risk factors considered. Individual studies were categorized into six broad groups according to the main risk factors presented. In Table 2, we provide a summary of the findings of each individual study, as well as the type of analysis (e.g., logistic regression), outcome measure, covariates, unadjusted and adjusted results, and quality rating. Statistical outputs in the main included unadjusted and adjusted ratios and risk, including the odds ratio (OR, aOR), relative risk (RR, aRR), hazard ratio (HR, aHR), and population-attributable risk percent (PAR). Estimates and their 95% confidence intervals were presented where possible. One study only presented results using a relative risk ratio (RRR, aRRR) which is the ratio of two relative risks (29). Supplementary Table 3 provides further details for each study, including exposures, covariates, and category of ID. Table 3 presents data according to individual risk factor rather than by study, providing the opportunity to compare qualitatively across studies and provide a summary of the findings. This presentation style was adopted as a quantitative synthesis (or meta-analysis) was not feasible given the heterogeneity of the findings.

**TABLE 1:** Characteristics of included studies (*n* = 58).

References Location	Type of Study	Data source (Population/cohort exclusions)	Time period	Sample size Number with ID	Comparator group	Classification criteria (exclusion for ID group)	Risk factors/Exposures considered
**Sociodemographic studies**							
Abdullahi et al. (29) Western Australia, Australia	Population-based birth cohort (with data linkage)	Midwives Notification System (MNS), WA Intellectual (IDEA) Database, WA Register of Developmental anomalies. Exclusion: multiple births.	Live births 1980–2010.	764,769 Number with ID: 10,625	Remainder of population	ID with or without ASD and CP. ID criteria = IQ<70.	Mothers’ country of birth, foreign born mothers from low-income, lower-middle-income, and upper-middle-income countries, Australian Indigenous mothers.
Camp et al. (83) United States	Prospective cohort (no data linkage)	Collaborative Perinatal Project.	1994 birth cohort followed to 2002. Age at assessment: 7 years.	Total 35,704 Number with ID: 1,311	Total population. 17,432 White children and 18,538 Black children.	IQ <70 as measured on the WISC.	12 postnatal variables divided into two tiers based on the frequency of coexistence with ID.
Chapman et al. (82) Florida, United States	Population-based birth cohort (with data linkage)	Florida public school and birth records.	Born 1992–1994, diagnosed in public school system 1996–1997.	267,277 Number with ID: 5,241	Remainder of population	Students aged 12–15 years: educable mentally handicapped (EMH), trainable mentally handicapped (TMH), or profoundly mentally handicapped (PMH).	Maternal age and maternal education
Decoufle et al. (78) Atlanta, United States	Case control (no data linkage)	Metropolitan Atlanta Developmental Disabilities Study (MADDS)	Born 1975–1977. Age at assessment: 10 years.	3,970 Number with ID: 352	408 public school students, aged 10, not receiving special education services.	Diagnosis of mild or severe ID on most recent standardised cognitive assessment. Inclusion: any ethnicity, born in 1975 or 1976 to a resident of the study area; and resident at age 10.	Maternal employment in a particular exposure category.
Decoufle and Boyle (79) Atlanta, United States	Case control (no data linkage)	Metropolitan Atlanta Developmental Disabilities Study (MADDS)	Born 1975–1977	1,018 Number with ID: 526	Public school students not receiving special education services.	Diagnosis of ID (<70) on most recent standardised cognitive assessment; excluded ID with biomedical cause.	Maternal education at time of delivery.
Drews et al. (75) United States	Case control (no data linkage)	Metropolitan Atlanta Developmental Disabilities Study (MADDS)	Born 1975–1977. 10-year-old children born in metropolitan Atlanta and living there in 1985 or 1986.	1,021 Number with ID: 458 (316 no known other neurological condition).	563 public school controls, aged 10, in Atlanta area in 1985 or 1986: *n* = 563.	ID = IQ <70. Public school records and health and social service agencies. Included known other neurological conditions; isolated ID without known neurological conditions.	Sociodemographic characteristics: sex, maternal age, birth order, race, economic status, and maternal education.
Emerson (74) England	Cross-sectional (with data linkage)	English Spring School Census, Department of Education	2008. Age at assessment: 7–15 years.	5,180,550 Number with ID: 248 628 (4.8%)	General population	Moderate learning difficulties (mild ID), severe learning difficulties (moderate ID) and profound multiple learning difficulties (severe ID).	Household disadvantage, local area deprivation, and ethnicity.
Heikura et al. (69) Finland	Birth cohort (no data linkage)	Questionnaires returned by the mothers during the 25th week of gestation. Follow-up to 11 years.	1966 and 1986, 2 cohorts 20 years apart. Age at assessment: 11.5 years.	1966 (12,058), 1986 (9,432) Number with ID: 1966 = 151; 1986 = 119.	General population	Standardized psychometric tests administered by a psychologist with Finnish ICD-9 or on a clinical basis evident child had ID.	Maternal sociodemographic factors assessed during pregnancy; change over 20-year interval.
Leonard et al. (60) Western Australia, Australia	Population-based birth cohort (with data linkage)	Maternal and Child Health Research Database (MNS).	Born 1983–1992	Total 239,829 Number with ID: 2871.	Remainder of unaffected population: 236,974.	IQ < 70 on formal testing Exclusions: Biomedical cause of ID.	Infant characteristics: sex and birth order. Parental characteristics: ethnicity, age, marital status, height, country of birth, health insurance status, socioeconomic status, geographical remoteness, paternal occupation.
Leonard et al. (62) Western Australia, Australia	Population-based birth cohort (with data linkage)	Singleton births, MNS and IDEA Database.	Born 1984–1999. Age at assessment: >6 years.	Total 398,353 Number with ID: 4,576	Remainder of singletons born in WA between 1984 and 1999 and alive in 2005 (*n* = 376,529).	ID identified as assessed on psychometric assessments or equivalent. Exclusions: Biomedical cause of ID.	Infant characteristics: sex and birth order. Maternal ethnicity, marital status, height, country of birth and maternal and paternal age at birth, socioeconomic status and geographical remoteness.
Oswald et al. (43) United States	Cross-sectional (with data linkage)	U.S. Department of Education Office for Civil Rights biennial collection of data.	School enrolments 1994–1995.	41,819,191 Number with ID: 577,360	Remainder of cohort: 38,645,020.	ID included students with mild, moderate and severe ID grouped together; classification method not defined.	District-level sociodemographic variables; child sex and ethnicity.
Rantakallio (42) Northern Finland	Population-based birth cohort (no data linkage)	Birth certificates from two provinces in Finland, Oulu and Lapland.	1966 births, followed up in 1980 and 1981.	12,058 live births Number with ID: 164	General population	IQ < 70 on psychometric testing. Exclusions: biomedical cause of ID or known risk factor.	Social class.
Stromme and Magnus (38) Norway	Cross-sectional (no data linkage)	Children living in Akershus County on 1 January, 1993.	Born 1980–1985. Age at assessment 3–13 years.	30,037 live births Number with ID: 178	General population	IQ <70, from IQ test, a standardized psychometric test, or on formal developmental assessment.	Socioeconomic status grouped by parental education.
Williams and Decoufle (33) Atlanta, United States	Case control (no data linkage)	Metropolitan Atlanta Developmental Disabilities Study (MADDS)	1985–1987. Age at assessment: 10 years.	1,255. Number with ID: 682	573 randomly selected public school control children.	10 year old children with ID (IQ < 70) from multiple sources.	Advanced or very young maternal age at delivery.
Yeargin-Allsopp et al. (32) Atlanta, United States	Case control (no data linkage)	Metropolitan Atlanta Developmental Disabilities Study (MADDS)	Cohort born in 1975 or 1976 in Atlanta study area, living there at age 10.	893 Number with ID: 330	563 randomly selected public school control children in metropolitan Atlanta area in 1985 or 1986.	Mild ID (IQ 50–70) on most recent psychometric examination	Race, median family income.
Zhen et al. (31) United States	Pregnancy cohort (with data linkage)	Medicaid inpatient and outpatient reimbursement files, birth certificate data, and hospital and outpatient care of pregnant women.	1996–2001. Age at assessment: 5–10 years.	22,429 mother child pairs Number with ID: 792	Population comparison group	Diagnosis of ICD9 317,318 or 319 in the Medicaid file Exclusions: Biomedical cause for ID	Spatial analysis of ID and maternal residence during pregnancy.
Zheng et al. (30) China	Cross-sectional (no data linkage). Stratified multiphase and cluster probability sampling.	Second National Sampling Survey on Disability in China	1/5/2006–30/5/2006. Age at assessment: up to 6 years.	106,774 Number with ID: 1308	Surveyed children not with ID.	Diagnosis using Gesell Developmental Inventory or Japanese version of Vineland Social Maturity Scale-for analysis classifies as mild (DQ55-75) or severe (DQ < 55).	Child’s age, sex, residence, geographic region, mother’s age group, maternal age group, family income, parental ID
**Antenatal and perinatal**							
Bilder et al. (85) Utah, United States	Case control (no data linkage)	All 8-year old children born in Utah’s three most populous counties (Salt Lake, Davis and Utah County).	Children born in 1994, studied in 2002. Age at assessment: 8 years.	17,082 Number with ID: 146 within study area (inclusive group). Second analysis with only children without a known or suspected genetic disorder (*n* = 115).	Matched by birth year and gender to 116 controls without ID or ASD: (9,976 males and 6,960 females); total of 16,936 controls.	IQ <70, from most recent IQ test or a statement on child’s functioning level as being in the ID range.	Information regarding 24 potential prenatal and perinatal risk factors was obtained from birth certificate records.
Chen et al. (80) Sweden	Population-based birth cohort (with data linkage)	Medical Birth Register, Patient Register, Cause of Death Register, Education Register, Total Population Register, Multi-Generation Register Exclusion: multiple births, any major malformations	Infants born AGA at term or post-term, between 1998 and 2009. Age at assessment: >3 years.	828,948 non-malformed term or post-term AGA singleton children (including 429,379 full siblings) Number with ID: 1,688	Same cohort without ID.	ID defined through hospital contact, with clinical diagnosis of ICD-10 codes F70-F79 from the Patient Register.	Birth weight for gestational age percentile and gestational age.
Chen et al. (81) United States	Prospective cohort (no data linkage)	US Collaborative Perinatal Project (CPP). 12 hospitals in the United States, placenta taken after birth. Exclusions: multiple births	Born 1959–1976. Ages at assessment: 8 months, 4 and 7 years.	32,326. 7,782 (24.1%) women had positive placental inflammatory pathology. Number with ID: at 4 years: 1,094 (of 25,611); at 7 years: 876 (of 26,827)	Same cohort without ID.	The CPP conducted neurologic and psychological examinations. The Stanford-Binet IQ scale assessed IQ at 4 years. At age 7 used the WISC. ID as IQ < 70.	Placental inflammation (placentas examined by pathologists blinded to clinical conditions). 10 measures specific to histologic chorioamnionitis.
Croen et al. (13) California, United States	Population-based birth cohort (with data linkage)	Service agency records (California Department of Developmental Services, DDS), birth certificates.	Children born in California 1987–1994, mothers were Californian residents at the time of delivery.	Total 4590333 Number with ID: 16,735	Californian births during the same period	ID via California DDS, defined as “significantly subaverage intellectual functioning, existing concurrently with related limitations in at least 2 adaptive skill areas, and manifesting before age 18”. Exclusions: Biomedical cause of ID	Infant and maternal characteristics of children with mild and severe ID of unknown cause.
Heuvelman et al. (68) Sweden	Population-based birth cohort (with data linkage)	Stockholm Youth Cohort; examined associations in a nested cohort of matched outcome discordant siblings. Exclusions: multiple births, adoptees, improbable birth weights <350 g or >6,000 g.	2000–2011 (Born 1984–2011). Age at assessment: >4 years.	499,621 Number with ID: 8,034	Discordant siblings	Used 4 registers to identify all inpatient or outpatient diagnoses of ID recorded using ICD-10 (F70-79) and DSM-IV (317–318) codes and supplemented with a record of care. Exclusions: genetic and inborn metabolic syndromes who had been diagnosed with ID (13.6% of cases).	Exposure: gestational age, with very preterm (21–31 completed weeks), moderately to late preterm (32–36 weeks), term (37–41 weeks), post-term (42 weeks) and very post-term births (43–45 weeks).
Jones et al. (65) California, United States	Case control (no data linkage)	Early Markers of Autism Study. Participant in the prenatal extended alpha-fetoprotein screening program (XAFP) in Orange, or Imperial Counties.	Deliveries from July 2000 to September 2003 in California.	Total 1,051 (including cases with ASD) Number with ID: 188	General population randomly sampled from birth records matched by sex, birth month and year: 428	ASD or ID ascertained from regional centers operated by the California Department of Developmental Services (DDS). ID = composite score < 70, DD defined as ID without ASD or Trisomy 21.	Mid-gestational maternal cytokine and chemokine levels
Langridge et al. (64) Western Australia, Australia	Population-based birth cohort (with data linkage)	MNS, Birth and Death Registers and IDEA Database.	Born January 1984 to December 1999. Age at assessment: <6 years.	Total population 383,153 Number with ID: (without ASD): 4,576 Number with ID (with ASD): 727	Remainder of children born 1984 to 1999, alive in 2005 and not identified as having ID or ASD: 376,539.	ID (IQ < 70) identified from the IDEA Database, as assessed on psychometric assessments or equivalent Exclusion: biomedical cause of ID.	Maternal conditions and perinatal factors (sociodemographic factors, labor and delivery characteristics and neonatal outcomes)
Leonard et al. (61) Western Australia, Australia	Population-based birth cohort (with data linkage)	MNS and IDEA Database.	Born 1983–1992. Age at assessment: >10 years.	Total 240,351 Number with ID: 2,625	Remainder of non-ID population: 217,252	ID (IQ < 70) identified from the IDEA Database, as assessed on psychometric assessments or equivalent Exclusion: cases with a biomedical cause.	Intrauterine growth (percentage of optimal birth weight, which accounts for gestational age, maternal height, parity, and infant sex).
Louhiala (58) Central and Southern Finland	Case control (with data linkage)	Perinatal data collected from 12 maternity hospitals in the area.	1967–1981	Total 189,051 Number with ID: 339	Control cases randomly selected from hospital delivery book of same birth year as case child.	ID (IQ < 71). Exclusions: Biomedical cause of ID.	Maternal demographics, pregnancy and birth characteristics.
Luu et al. (57) Portland and New Haven, United States	Case control (no data linkage)	375 children born 1989-1992 with birth weight 600-1,250 g enrolled in the Indomethacin Intraventricular Hemorrhage (IVH) Prevention Trial.	1989–1992. Age at assessment: 12 years.	375 cases and 111 controls Number with ID: 52	Term controls from telemarketing list, frequency-matched to PTB group on zip-code, age, gender, maternal education, and race: 111	Cognitive functioning with WISC-III.	Preterm birth <1,250 g. Considered impact of neonatal brain injury, indomethacin and environmental risk factors on ID at 12 years.
McDermott et al. (50) California, United States	Cohort study (no data linkage)	Subgroup of the Child Health and Development Studies (CHDS) data, mothers insured by Kaiser Health	Children born 1964–1966 assessed at age 5 years. Children born 1960–1963 Age at assessment: 9–11 years.	5-year old cohort, number with ID: 159. 9–11 year olds, number with ID: 215.	5-year-old cohort: total 3,001 children. 9–11-year-olds: total 3,293 children.	Scores of 50–70 on the Raven Progressive Matrices = ID.	Low birth weight: <2,500 g. Intermediate birth weight: 2501–2,954 g, reference normal birth weight >2,955 g.
Mervis et al. (47) Atlanta, United States	Case control (no data linkage)	Metropolitan Atlanta Developmental Disabilities Study (MADDS)	1975–1996. Age at assessment: 7–10 years.	Total 1,014 Number with ID: 441	10 year old public school control children randomly chosen from education files: 573	IQ < 70 on most recent psychometric assessment	Birth weight
Schieve et al. (40) United States	Cohort (with data linkage)	Children in the 2006–2010 Autism and Developmental Disability Monitoring Network (ADDM) Exclusion: multiple births.	Born 1998, 2000 and 2002. Age at assessment: 8 years	Total 7,547 (including 2726 with ASD no ID) Number with ID: 4821	Compared with expected numbers in general population, excluding infant deaths before age 1.	IQ < 70 as measured on standardized IQ assessment. Classifications: ASD + ID, ASD only, and ID only.	Pre-term birth, low birth weight, small for gestational age, and low Apgar score
Van Naarden et al. (35) United States	Cohort (with data linkage)	ADDM Network, 2002 surveillance year data files across 11 sites.	Born 1994. Age at assessment: 8 years.	Total: ASD (51,626; 50 born as part of an MB); CP (5,302; 25 born as part of an MB); or ID (51,195; 45 born as part of an MB). Number with ID: 51,195	General population (national natality files), 1971 and 1994	ID (IQ < 70) on the most recent psychometric test or a written statement by a psychometrist that a child’s intellectual functioning is within the range for severe or profound ID.	Multiple births
**Maternal physical health**							
Blotiere et al. (84) France	Population-based birth cohort (with data linkage)	The French national health insurance, and hospital discharge databases, singleton pregnancies.	Children born alive 2011–2014, followed from birth until death, loss to follow up or to 31 December 2016, whichever came first.	9,034 children, 2,916 exposed to lamotrigine, 1,627 pregabalin, 1,246 clonazepam, 991 valproic acid, 621 levetiracetam, 502 carbamazepine, 477 topiramate, 378 gabapentin 143 oxcarbazepine. Exclusion: brain malformation. Number with ID: 45	Pregnant women exposed to lamotrigine monotherapy	The primary outcomes were hospitalization or insurance for neurodevelopmental disorders (ICD-10 diagnosis codes F70–F98), and for two specific subcategories: pervasive developmental disorders (F84) and ID (F70–F79).	Antiepileptic drugs (AED) compared with lamotrigine. Exposed defined as 30 days following dispensing of an AED. Monotherapy defined as the absence of any other AED dispensed during the same period.
Drews et al. (76) Atlanta, United States	Case control (no data linkage)	Metropolitan Atlanta Developmental Disabilities Study (MADDS)	Born 1975–1976	Total 621. Number with ID: 221	Mothers of aged 10 public school children born in study areas in 1975 or 1976 and living there.	Children with ID (IQ < 70) on the most recent psychometric test. Exclusions: Biomedical cause of ID.	Maternal smoking during pregnancy. Data obtained through interviews with mothers.
Griffith et al. (71) South Carolina United States	Retrospective birth cohort (with data linkage)	Medicaid births excluding multiples 1996 to 2002 linked to Medicaid records, delivery records, birth certificates, DE and DDSN.	1996–2002. Age at assessment: 5 years in public schools, 3 years for those with ID.	80,866 mother-child dyads. Number with ID: 1,636	Remainder of unaffected population.	Special education in public system or ID related services from DDSN. Exclusions: Biomedical cause of ID	Maternal preeclampsia and eclampsia, and low birth weight.
Leonard et al. (63) Western Australia, Australia	Population-based cohort (with data linkage)	Disability Services Commission (DSC) (birth to age 16), educational sources (aged 7–16 years)	Born 1983 to 1992, identified with ID by 1999 and alive in 2002.	Total population 239,829. Number with ID: 2,865 (mild-moderate *n* = 2,462; unspecified *n* = 361; severe or profound ID *n* = 212; ASD with ID *n* = 191).	Remainder of non-ID population: *n* = 236,964	Depending on test type, ID identified through DSC or education source: mild to moderate IQ 35/40–69, severe (including profound) ID, IQ < 35 or 40.	Asthma, diabetes mellitus, undiagnosed cardiac murmur, hypertension, renal and urinary conditions, epilepsy, anemia, genital herpes, infertility, hepatitis, hypothyroidism, aggregated socioeconomic status measure.
Li et al. (59) Boston, United States	Cohort (no data linkage), 1:2 matched design: per 1 preterm or LBW, 2 term and 2 normal BW subjects.	Subset of Boston Birth cohort with at least one post-natal follow-up visit 1998 - 2014. Excluded multiple births.	1998–2014. Age at assessment: range 3.6–9 years (median 6 years)	Total 2,734. Number with ID: 137.	Remainder of population without ID, DD, ASD or ADHD.	Classification for ID (317, 318.0–318.2, and 319), and/or Down syndrome (758.0)	Maternal pre-pregnancy height and weight (obesity), maternal diabetes
Mann et al. (55) South Carolina, United States	Retrospective birth cohort (with data linkage)	Medicaid reimbursement files, birth certificates, school and disability records Jan 1996-March 2011	Born 1996–2002	134,596 mother-child pairs after exclusions Number with ID: 5,388	Those without ID in same cohort.	3 data sources: ICD-9 codes for ID in Medicaid file; or categorisation of ID from the DOE; or enrolment in ID support services (required two formal psychological assessments). Exclusions: biomedical cause of ID.	Maternal trichomoniasis (4023 cases) and GU infections identified using ICD 9. Treatment status using Medicaid outpatient pharmacy billing records.
Mann et al. (54) South Carolina United States	Retrospective birth cohort (with data linkage)	Medicaid billing birth certificates and delivery records from births 2004–2007, linked to data from the South Carolina Department of Education (DOE) & the South Carolina Department of Disabilities and Special Needs (DDSN).	2004–2007. Age at assessment: >3 years.	78,675 mother child pairs Number with ID: 3,113	Those without ID in same cohort.	3 data sources: ICD-9 codes for ID in Medicaid file; or categorisation of ID from the DOE; or enrolment in ID support services (required two formal psychological assessments). Exclusions: biomedical cause of ID.	Maternal pre-pregnancy BMI (based on height and weight), weight change during pregnancy
Mann et al. (53) South Carolina, United States	Retrospective birth cohort (with data linkage)	Medicaid claims, DOE, and DDSN data 2000–2007; minimum follow-up 3-years post birth or until a diagnosis of ID.	2000–2007. Age at assessment: >3 years.	165,311 (70.97 %) eligible pairs Number with ID: 5,780	Those without ID in same cohort.	ID identified as a case in at least one of the three data sources. DOE/DDSN data or ICD9-CM code (317–319) for ID in the Medicaid billing records on >5 occasions. Exclusions: Biomedical cause of ID.	Maternal diabetes mellitus
McDermott et al. (51) South Carolina, United States	Prospective cohort	Matched maternal-child pairs from the US National Collaborative Perinatal Project (NCPP), compared with previous analysis of South Carolina Medicaid data 1995–1996.	NCPP 1958–1974. Assessed at 8 months, 4 and 7 years	41,692 mother-child pairs. 37,669 no ID. Number with ID: 1,760.	Medicaid data set from 1994 to 1996	Standardized scores on the Stanford-Binet Intelligence Scale Form LM, at age 4 years in the NCPP data set, with IQ < 70 classified as ID.	Maternal UTI during pregnancy.
McDermott et al. (49) South Carolina, United States	Retrospective birth cohort (with data linkage)	Birth certificate data, hospital discharge data and Medicaid prenatal and maternity care claims.	Born 1995–1998.	41,090 mother-child pairs. Number with ID: 617	Medicaid data set from 1994 to 1996	ICD-9 codes for ID in the Medicaid file. Exclusions: known causes of ID.	Women with UTI during pregnancy with/without antibiotic claim. Includes Medicaid claim (ICD-9-CM diagnosis) or urinalysis and antibiotic claim.
Salonen and Heinonen (41) Finland	Case control single Finnish county	ID cases selected from local developmental defect registries.	Age at assessment: 9–10 years in 1979–1981.	Total 258 Number with ID: 136	Same area, same age in general population: 122 controls.	Defined as severe or mild ID from records or screening.	Maternal hypertension during pregnancy
Takei et al. (37) United Kingdom	Birth cohort (no data linkage)	Live births in England and Wales per month 1953 to 1980 from the Office of Population Censuses and Surveys.	Births 1953–1980, discharged 1976–1986.	827 first admission individuals (mean age 13 years) with a primary diagnosis of non-specific ID, discharged from psychiatric hospitals in England and Wales.	Those not developing ID.	ICD-8 primary diagnosis of ID (ID; 310–315) or ICD-9 (317–319), *n* = 827:478 males and 349 females). Exclusions: known biomedical cause of ID.	Number of pregnant women exposed to influenza was the total number of female deaths attributed to influenza in England and Wales each month between 1952 and 1980.
Tomson et al. (36) Sweden	Population-based birth cohort (with data linkage and propensity scores)	Swedish Medical Birth Register, National Patient Register, Prescribed Drug Registry, Education Register.	Singleton live births at=22 weeks GA 2006–2016.	4,544 births to 2,955 fathers with epilepsy, of which 2087 (45.9%) born to fathers who had dispensed an AED during the conception period. Number with ID: 4,350.	1 144 795 births to 741 726 fathers without epilepsy	Paternal epilepsy restricted to individuals whose epilepsy onset occurred before child’s birth, and those with active epilepsy (>10 years) (ICD-10 code G40) Clinically ascertained ID diagnoses identified from birth to end 2017 in the National Patient Register (ICD-10 codes F70-F73, F78, F79, R620).	Paternal AED exposure. Prescription medications coded using Drug ID Numbers and the Anatomical Therapeutic Chemical classification system. Men exposed to AED within 74 days prior to or at the time of conception. Maternal AED use was 30 days before the estimated day of conception to the day of birth.
**Maternal mental health**							
Di Prinzio et al. (77) Western Australia, Australia	Population-based birth cohort (with data linkage)	Birth Register, Midwives Notification system, Mental Health Information System, IDEA Database.	1980–2001. Age at assessment: <6 years	6,303 children born to mothers with (3,174) and without (3,129) psychiatric illness. Number with ID: 129	Random sample of children of born 1980–1992 to mothers unaffected by psychiatric illness: 3129.	ID identified as assessed on psychometric assessments or equivalent. through WA IDEA Database	Maternal psychiatric disorders (schizophrenia, bipolar disorder, unipolar major depression, paranoid states, and other nonorganic psychoses).
Fairthorne et al. (72) Western Australia, Australia	Population based birth cohort (with data linkage)	Birth Register, Midwives Notification system, Mental Health Information System, IDEA Database.	Child born in WA between 1983 and 1999 inclusive	213,656 mothers. 9,341 (4.4%) with psychiatric record. Number of ID of unknown cause: 3860 (severe ID: 254); ID of known cause: 926.	Mothers without child with ASD or ID, index child was the eldest child born 1983 to 1999. *n* = 207,827	ID identified from the IDEA Database, as assessed on psychometric assessments or equivalent	Women who received a psychiatric diagnosis had their contact and diagnosis recorded in the MHIS dataset.
Morgan et al. (46) Western Australia, Australia	Population-based birth cohort (with data linkage)	Birth Register, Midwives Notification system, Mental Health Information System, IDEA Database.	Births 1980–1992. Age at assessment: >6 years.	3174 children born to mothers with schizophrenia, bipolar disorder or major depression. Number with ID: 129	Random sample of 3129 children of unaffected mothers.	ID identified from the IDEA Database, as assessed on psychometric assessments or equivalent; ID included borderline low IQ (70–74).	Maternal psychiatric disorders (schizophrenia, BPD and unipolar major depression).
O’Leary et al. (45) Western Australia, Australia	Population-based birth cohort (with data linkage)	WA MNS (1983–2001), hospital morbidity, mental health, drug and alcohol data sets linked to IDEA Database and Register of Developmental Anomalies	Born 1983–2001. Age at assessment: up to 6 years.	18,571 children (of case mothers 10,664, comparison mothers 7,907) Number with ID: 1,487	Mothers without an alcohol-related diagnosis, frequency-matched on age within maternal Aboriginal status and birth year of children.	ID (IQ < 70) identified from the IDEA Database, as assessed on psychometric assessments or equivalent Exclusion: biomedical cause of ID.	Maternal alcohol use disorder in pregnancy (alcohol-related diagnosis on any data set used as a proxy)
Wang et al. (34) South Carolina, United States	Retrospective birth cohort (with data linkage)	Medicaid billing birth certificates and delivery records from births 2004–2007, linked to DOE and DDSN. Exclusions: multiple births.	2004–2010. Age at assessment: >3 years.	123,922 Number with ID: 4,771	Those without ID in same cohort.	ID from 3 data sources: DOE/DDSN data or ICD9-CM code (317–319) for ID in Medicaid billing records on >5 occasions. Exclusions: Biomedical cause for ID	Mothers with schizophrenia, bipolar disorder, Unipolar Major Depression or mental disorders complicating pregnancy.
**Environmental**							
Emerson et al. (73) United Kingdom	Birth cohort (with data linkage)	Waves 1–6 of UK Millennium Cohort Study. Children assessed at 9 months, 3, 5, 7, 11, and 14 years.	UK children born September 2000 to January 2002.	9 months: ID 552 3 years: ID 525 5 years: ID 528 7 years: ID 489 11 years: ID 556 14 years: ID 432	9 months: 18,000 3 years: 14,371 5 years: 14,384 7 years: 12,760 11 years: 15,063 14 years: 10,885	Children with IQ scores 2+ SDs below the mean identified from testing at each age. For 125 children where there were no cognitive test results, ID was identified via parental reports.	Annual mean values air pollutants from data linkage through residential postcode with air pollution data: diesel particulate matter, nitrogen dioxide, sulfur dioxide and carbon monoxide.
Mackay et al. (56) Scotland	Population-based birth cohort (with data linkage)	Scotland-wide record linkage of education (annual pupil census) and maternity (Scottish Morbidity Record 02) databases. Exclusions: multiple births.	2011 Scottish census. At school 2006- 2012. Age at assessment: 4–19 years.	Total 801,592 (734,806 controls, 66,786 special needs) Number with ID: 17,942	From birth cohort without special education needs: 734,806	Special education needs attributed to ID.	Seasonality (month of conception of child with ID).
McDermott et al. (48) South Carolina United States	Retrospective birth cohort (with data linkage)	Medicaid reimbursement files, birth certificates, and hospital and outpatient care of pregnant women residing in one of six residential study strips.	1996–2002. Age at assessment: >8 years. 8–12 years of follow-up time	Total 3,988 mother child pairs (after exclusions) Number with ID: 246	Those without ID in same cohort: 3879.	ICD9-CM code (317–319) for ID in the Medicaid billing records. Exclusions: Biomedical cause of ID	The soil was sampled and analyzed for levels of arsenic, barium, chromium, copper, lead, manganese, nickel, and mercury.
McDermott (52) United States	Retrospective pregnancy cohort (with data linkage)	Retrospective cohort 1996 to 2002, mothers residing in one of ten areas (9 case areas, 1 control) during the 6th month of pregnancy.	1996–2002. Age at assessment: >9 years	Total 8,743 Number with ID: 514	Those without ID in same cohort: 8229.	At least two diagnosis of ICD9 317–319 in the Medicaid file or eligibility for special education support. ID based on actual IQ and adaptive test scores.	Soil concentrations of arsenic, barium, chromium, copper, lead, manganese, nickel and mercury in the soil proximal to maternal residence during pregnancy.
Onicescu et al. (44) South Carolina, United States	Retrospective pregnancy cohort (with data linkage)	Prenatal and pregnancy Medicaid claims data linked to birth certificates, school and disability records of women living in nine areas.	Born 1996–2002	7,279 Number with ID: 420	Those without ID in same cohort. *n* = 6859	ICD9-CM code (317–319) for ID in the Medicaid billing records or had a special education placement for ID. Exclusions: Biomedical cause of ID.	Concentration of soil levels of arsenic, chromium, mercury, lead, manganese, barium, copper and nickel at mothers’ residence during pregnancy.
**Genetic or biological**							
Guo et al. (70) Western China	Case control (no data linkage)	Recruited from Zha Shui and An Kang counties, Western China.	Age at assessment: 4–16 years.	Total 543 Number with ID: 212	Controls from the same iodine-deficient areas with no family history of ID: *n* = 331	Screened using the Adaptive Scale of Infant and Children and IQ assessed using the C-WYCSI or the C-WISC with cut-off of < 70 for ID.	Three polymorphisms in the DIO2 gene (rs225014, rs225012, and rs225010)
Hurtado et al. (67) Florida, United States	Population-based birth cohort (with data linkage)	Linked birth and school records, and early childhood nutrition data from the Special Supplemental Program for Women, Infants, and Children up to age 5 years.	Births 1979–1980, followed up 1990–1991. Age at assessment: 10 years.	5,411 across 3 data sources; complete data available for 3,771. Number with ID: 108	Children who were achieving normally in general population.	Criteria used by the Florida Department of Education for special education placement. Mild or moderate ID (EMH or TMH).	Mother and child demographics, early childhood anemia or low hemoglobin concentrations.
Jelliffe-Pawlowski et al. (66) California, United States	Population-based birth cohort (with data linkage)	Infants born in non-military hospitals in selected areas of California in 1992–1993 and who survived to 1 year of age. Linked to the California Birth Defects Monitoring Program and the DDS.	Born 1992–1993. Age at assessment: 7 years.	Total (1 year survivors) 119,556. Infants with (*n* = 2,337) or without (*n* = 117,219) birth defects. Number with ID: 591	Cases born in same period without a registered birth defect: 117,219.	Diagnosis of ID by age 7 years without other developmental disabilities (isolated ID) or as with cerebral palsy, epilepsy, or PDD. Exclusions: birth defect of external cause.	Birth defects, and individual and maternal factors.
Shaw et al. (39) California, United States	Case control (no data linkage)	Infants born in non-military hospitals in selected areas of California in 1992–1993 and who survived to 1 year of age.	Born 1992–1993	Total 843 Number with ID: 285	Control non-malformed infants randomly selected born in California 1987–1991: 743	213 children with IQ < 50; 72 children with adaptive functioning criteria for severe ID. Exclusions: normal intellectual functioning prior to severe head trauma, near drowning, or stroke.	Infants with homozygous genotype TT of the MTHFR gene.

MNS, Midwives Notification System (Western Australia, Australia); IDEA, Intellectual Disability Exploring Answers (Western Australia, Australia); ID, Intellectual Disability; EMH, Educable Mentally Handicapped; TMH, Trainable Mentally Handicapped; PMH, Profoundly Mentally Handicapped (Florida); MADDS, Metropolitan Atlanta Developmental Disabilities Study (Atlanta); ICD, International Classification of Disease; CPP, Collaborative Perinatal Project (United States); DDS, Department of Developmental Services (California); DSM, Diagnostic and Statistical Manual; ADDM, Autism and Developmental Disability Monitoring Network (United States); AED, Antiepileptic drugs; DSC, Disability Services Commission (Western Australia, Australia); DOE, Department of Education (South Carolina); DDSN, Department of Disabilities and Special Needs (South Carolina).

**TABLE 2:** Outcomes of included studies.

Study	Analysis type, Outcome measures	Covariates	Summary of Findings	Results (unadjusted OR or equivalent)	Results (adjusted OR or equivalent)	Quality rating*
**Sociodemographic**						
Abdullahi et al. (29) Western Australia, Australia	Logistic regression Odds ratios (OR) Relative risk ratios (RRR)	Proportion of optimal birth weight (POBW), gestational age (GA), sex, socioeconomic status (SES), birth year, maternal age, group, maternal medical conditions, smoking.	Children born to mothers from foreign-born low-income countries had an increased relative risk of autism spectrum disorder (ASD) with intellectual disability (ID), and children born to foreign-born mothers from upper-middle-income countries had an increased risk of cerebral palsy (CP) with ID.		Indigenous mothers: risk ID [aRRR 1.75 (1.60–1.92)] and CP with ID [aRRR 1.74 (1.25–2.41)]. Foreign-born low-income mothers: risk ASD with ID [aRRR 2.34 (0.96–5.70)]. Upper-middle- income countries: risk ASD with ID [aRRR, 1.12 (0.70–1.78)].	Med
Camp et al. (83) United States	Multivariate regression Relative Risk (RR) Chi-squares Population attributable risks (PAR)	Race (Black and White	Low SES of the family accounted for 44–50% of ID and a low level of maternal education accounted for 20%.	*Risk of ID for Black Children:* Low SES RR 2.54, PAR 49.95% OR Low maternal education RR 2.34, PAR 20.06%. *Risk of ID for White Children:* Low SES RR 3.36, PAR 43.94% Low maternal education RR 3.00, PAR 19.65%.		Med
Chapman et al. (82) Florida, United States	Risk ratio, Population attributable fraction	None	Low maternal education increased risk for all ID. Older maternal age increased risk of all ID, but for individuals with mild ID, this age effect was only seen in the lowest education group.	Mothers with <12 years education vs. some post-secondary education: RRR 7.00 (6.40–7.70).		Med
Decoufle et al. (78) Atlanta, United States	Multiple logistic regression Exposure odds ratios	Child race, maternal education level and birth order.	General pattern of lower than expected risk of ID in children of white collar workers and higher risk in children of blue collar workers.		Risk of any ID in children of blue-collar workers in the textile products or apparel manufacturing industries: aOR 10.30 (1.30–81.70).	High
Decoufle and Boyle (79) Atlanta, United States	Multiple logistic regression Odds ratios	Maternal race, age at delivery, child gender birth weight (BW), birth order, family SES.	Lower maternal education, race-education interaction. Maternal educational level strongly and inversely related to idiopathic ID.		Child risk of any ID for Black mothers with <10 years education: aOR 2.90 (1.60–5.40); White mothers: aOR 9.10 (3.90–21.30). Maternal education overall aOR 0.71 (0.65–0.79) Maternal education (White and Black combined) < 8 years aOR 6.10 (2.20–16.60) vs. > 16 years education aOR 0.20 (0.10–0.50)	High
Drews et al. (75) United States	Logistic regression Odds ratios	Models included all 6 study variables.	Boys, children with two or more older siblings, Black children, and children whose mothers had not completed high school were more likely to have both mild and severe ID than were girls, firstborn children, White children, and children with college-educated mothers. Older maternal age was associated with an increased prevalence of severe ID; low SES with mild ID. ID with no biomedical cause was associated with high birth order and low maternal education. Older maternal age was associated with an increased prevalence of ID with defined etiologies. Results held when dividing by IQ group (i.e., mild 50–70, severe < 50).		Mild ID (*n* = 330) Male sex: aOR 1.60 (1.20–2.20) Birth order > 3rd: aOR 1.60 (1.10–2.50) Black race: aOR 1.80 (1.30–2.60) Low SES: aOR 1.60 (1.00–2.50) Maternal education <12 years: aOR 4.10 (2.40–6.90) Maternal education = 12 years: aOR 1.80 (1.10–2.90) Severe ID (*n* = 128) Male sex: aOR 1.70 (1.10–2.50) Maternal age > 30 years: aOR 1.80 (1.10–3.10) Isolated ID (*n* = 316) Male sex: aOR 1.70 (1.20–2.30) Birth order > 3rd: aOR 1.90 (1.20–2.90) Black race: aOR 2.30 (1.60–3.30) Maternal education < 12 years: aOR 4.90 (2.80–8.50) Maternal education = 12 years: aOR 2.10 (1.20–3.40)	Med
Emerson (74) England	Multilevel logistic regression Odds ratio	Ethnicity, household deprivation and area deprivation.	Lower household SES was associated with increased likelihood of mild-moderate and severe ID but lesser likelihood for profound multiple ID. Higher rates of mild-moderate ID were found in Gypsy/Romany and Traveler children of Irish heritage and of severe iD in children of Pakistani heritage, Minority ethnic status was otherwise associated with lesser likelihood of ID.		Area deprivation codes d1-d9 aORs significant for mild ID (aOR 3.48 (3.38–3.57) at most deprived area) and severe ID (aOR 1.45 (1.36–1.55 at worst area).	Med
Heikura et al. (69) Northern Finland	Multivariate logistic regression Odds ratios	Maternal age, parity, body mass index, marital status, SES, and place of residence	Socioeconomic disadvantage and maternal multiparity had greatest impact on risk of ID. Pre-pregnancy maternal obesity became evident in the more recent cohort.	1966 cohort, risk for mild ID: SES IV Level 4 Unskilled OR 1.80 (0.60–5.30); Multiparity (parity > 4) OR 1.30 (0.70–2.60). 1986 cohort: SES IV Level 4 Unskilled OR 2.70 (1.50–4.70), Multiparity (parity > 4) OR 3.30 (1.80–6.00).	For any ID, in 1966 cohort: Multiparity (parity > 4) aOR 1.50 (0.60–3.60); 1986 cohort: Multiparity (parity=4) aOR 2.40 (1.20–4.90) Pre-pregnancy body mass index (BMI) aOR 2.80 (1.50–5.30) For any ID: 1966 cohort: SES IV Level 4 unskilled; aOR 1.70 (0.50–5.80). 1986 cohort: SES IV Level 4 Unskilled, aOR 2.20 (1.20–3.90) For mild ID, being a farmer in 1966 cohort aOR 3.50 (1.10–11.40); and in 1986 cohort aOR 3.70 (1.40–9.80). For severe ID, living in a remote area in 1966 cohort aOR 1.90 (1.00–3.60), level IV unskilled worker for 1986 cohort aOR 3.10 (1.40–6.70).	High
Leonard et al. (60) Western Australia, Australia	Logistic regression Odds ratios	Birth year	Aboriginal mothers, teenagers and single mothers at increased risk of having a child with mild or moderate ID. Children of mothers in the most socioeconomically disadvantaged 10% had more than five times the risk of mild and moderate ID than those in the least disadvantaged 10%. Fourth or later born children were also at increased risk.	Mild-moderate ID: Aboriginal mothers OR 2.83 (2.52–3.18), teenagers OR 2.09 (1.82–2.40) and single mothers 2.18 (1.97–2.42). Most socioeconomic disadvantaged 10%: OR 5.61 (4.40–7.12), 6th or later-born infants OR 3.13 (2.50–3.91).		Med
Leonard et al. (62) Western Australia, Australia	Multinomial logistic regression Odds ratios	Birth year, POBW, sex, birth order, maternal ethnicity, country of birth, height, marital status, maternal and paternal age, SES, geographical remoteness.	Gradient effect where risk factors for mild-moderate ID and ASD without ID were at opposite extremes. Mild-moderate ID was associated with younger mothers <20 years, paternal age >39 years, Australian-born and Aboriginal mothers, increasing birth order and increasing social disadvantage. Mothers of infants residing in regional or remote areas had consistently lower risk of ID.	Mild-moderate ID Maternal age <20 years, OR 2.41 (2.17–2.69); Paternal age <20 years, 2.38 (1.98–2.87); Aboriginal OR 1.79 (1.15–2.78); Low SES OR 2.56 (2.27–2.97; 4th. or later born OR 2.06 (1.89–2.25).	Mild-moderate ID Maternal age < 20 years, aOR 1.88 (1.57–2.25); paternal age >39 years, aOR 1.59 (1.36–1.86); Aboriginal aOR 1.60 (1.41–1.82; low SES aOR 2.56 (2.27–2.97); 4th or later born, aOR 2.55 (2.27–2.87).	Med
Oswald et al. (43) Columbia, United States	Logistic regression Odds ratios	District weights.	ID more prevalent in males but relationships with gender vary by racial group.	ID by Gender/Ethnicity Groups: Male American Indian: OR 1.66 Male Black: OR 3.26 Male White: OR 1.36 Female Black: OR 2.02	ID by Gender/Ethnicity Groups: Male American Indian: aOR 1.60 Male Black: aOR 4.03 Male Hispanic: aOR 1.35 Female Black: aOR 2.58	Med
Rantakallio (42) Northern Finland	Pearson chi square Proportions	None	Mild and severe ID associated with lower class and farmers.	Idiopathic ID Social class I & II Inc. mild ID 0.4 per 1,000 Inc. moderate ID 0.4/1,000 Social class IV Inc. mild ID 2.5/1,000 Inc. moderate ID 1.9/1,000		Low
Stromme and Magnus (38) Norway	Logistic regression Odds ratios	None	Lower SES was associated with mild ID. Severe ID associated with higher SES.	Children with parents in Social Classes IV to V had increased risk of mild-moderate ID compared with severe ID OR 6.30 (0.70–23.50) for IV OR 5.00 (1.50–15.60) for V		Med
Williams and Decoufle (33) Atlanta, United States	Logistic regression Odds ratios	Child gender, BW and birth order, maternal age, education, race, father presence.	Increased risk of biomedical ID was seen among Black children of mothers aged >30 years not attributable to Down syndrome	Biomedical ID: Maternal age <20 years: OR 2.82 (1.51–5.26) Idiopathic ID Maternal age <20 years: OR 1.39 (0.76–2.47)	Biomedical ID Maternal age <20 years: aOR 2.20 (1.06–4.59) Idiopathic ID Maternal age <20 years: aOR 0.85 (0.40–1.83)	High
Yeargin-Allsopp et al. (32) Atlanta, United States	Logistic regression Odds ratios	Sex, maternal age, birth order, maternal education, SES.	Black race, but not for children diagnosed before 6 years of age.	Risk of mild ID in Black compared with White children: OR 2.60 (2.00, 3.50)	Risk of mild ID in Black compared with White children: OR 1.80 (1.30–2.60)	High
Zhen et al. (31) United States	Bayesian local likelihood cluster modeling RRs Chi-square *p*-values	None	Using spatial analysis for cluster detection according to mother’s geocode during her pregnancy a of ID was higher than surrounding areas. The descriptive analysis of the study population showed that in general children with ID were more likely to be male and had mothers who were older than 34 years at the time of birth as well as being African American, preterm and of low birth weight compared to children without ID.	Rate per 100 for ID cases in study area in South Carolina (n), ID cases in cluster area (n) Month 1: 4.3 (1287), 5.9 (185). Month 2: 4.1 (2213), 7.0 (273) Month 3: 4.0 (2813), 8.0 (337) Month 4: 3.9 (3169), 8.5 (366) Month 5: 3.9 (3331), 7.7 (388) Month 6: 3.8 (3386), 7.6 (395) Month 7: 3.6 (3225), 7.1 (408) Month 8: 3.4 (2887), 7.0 (370) Month 9: 3.2 (2091), 5.3 (281) Month 10: 3.8 (523), 8.9 (79)		Low
Zheng et al. (30) China	Logistic regression Odds ratios	Age, gender, parent disability	Male sex, mother’s age, Illiteracy, rural residence, parental ID were risk factors for both mild and severe ID as. Family income lowest compared with highest showed a monotonic increase in the likelihood of ID.	Mild ID: Male OR 1.22 (1 04–1.42) Maternal age >34 vs. 20–29 OR 1.77 (1.35–2.31) Mother illiterate vs. Senior High School or higher OR 4.53 (3.63–5.66) Rural vs. Urban residence OR 2.21 (1.86–2.63) Parental ID vs. Non-ID OR 14.67 (10.74–20.05) Severe ID: Male OR 1.13 (0.96–1.32). Maternal age >34 vs. 20–29 OR 1.53 (1.14–2.05) Mother illiterate vs. Senior High School or higher: OR 5.67 (4.51–7.13) Rural vs. Urban residence OR 1.73 (1.44–2.09) Parental ID vs. Non-ID OR 13.81 (9.87–19.30)	Mild ID: Male vs. female aOR 1.23 (1 06–1.44) Maternal age > 34 vs. 20–29 aOR 1.47 (1.15–1.88) Mother illiterate vs. Senior High School or higher aOR 1.74 (1.21–2.52) Rural vs. Urban residence aOR 1.93 (1.55–2.41) Parental ID vs. Non-ID aOR 8.81 (6.45–12.06) Severe ID: Male vs. female aOR 1.14 (1.00–1.73). Maternal age > 34 vs. 20–29 aOR 1.32 (1.14–2.05) Mother illiterate vs. Senior High School or higher aOR 2.45 (1.65–3.63) Rural vs. Urban residence aOR 1.35 (1.09–1.67) Parental ID vs. Non-ID aOR 7.10 (5.09–9.91)	Med
**Antenatal and perinatal**						
Bilder et al. (85) Utah, United States	Logistical regression Multiple logistic regression.	Maternal and paternal age, education, GA and parity	Poly/oligohydramnios, advanced paternal/maternal age, prematurity, fetal distress, premature rupture of membranes, primary/repeat cesarean sections, low birth weight, assisted ventilation greater than 30 min, small-for-gestational age, low Apgar scores, and congenital infection, associated with increased odds of mild to severe ID.	*Risk of mild and severe ID* Maternal age > 34 OR 1.82 (1.20–2.76) Paternal age > 34 OR 2.00 (1.38–2.90) Uterine bleeding OR 2.63 (1.07–6.4) Poly/oligohydramnios OR 5.45 (2.51–11.84). Maternal education > 13 years OR 0.67 (0.47–0.96)	*Risk of mild and severe ID* GA < 37 weeks aOR 3.07 (1.98–4.76) Primary cesarean section aOR 3.95 (2.48–6.30) Premature rupture of membranes aOR 2.48 (1.20–5.13) Fetal distress aOR 3.05 (1.75–5.30) Low birth weight < 2500 gm aOR 3.43 (2.21–5.33) Low 5-min Apgar score < 7 aOR 8.11 (4.02–16.36) Assisted ventilation > 30 min aOR 5.35 (2.61–10.98)	High
Chen et al. (80) Sweden	Cox regression models. Hazard ratios (HR)	Maternal age, education parity, country of birth, smoking, height, weight, labor onset, mode of delivery, sex, birth period,	Among appropriate-for-gestational-age (AGA) children born at term or post-term, lower birth weight percentiles within the normal range are associated with increased risk of ID, regardless of GA.		Compared with the reference group (40th–59th birthweight percentiles): 10th–24th percentiles aHR 1.43 (1.22–1.67) (population analysis) aHR 1.52 (1.00–2.31) (sibling analysis) 25th–39th percentiles: aHR 1.28 (1.10–1.50) in (population analysis) aHR 1.44 (1.00–2.09) (sibling analysis)	High
Chen et al. (81) United States	Logistic regression. Odds ratios.	Model 1: maternal age, race, SES, education, pre-pregnancy BMI, smoking, parity, sex, GA.	Placental inflammation was associated with risk of ID at 7 years of age. No indirect effects of shorter GA.		Risk of ID at 7 years of age: aOR 1.20 (0.97–1.48)	Low
Croen et al. (13) California, United States	Poisson regression. Risk ratios. Adjusted RRs.	Gender, weight, plurality, parity, maternal age, race/ethnicity, education, birthplace.	For both children with mild and severe ID, risk was increased among those who were male, low birth weight, born to women of Black race, older age at delivery, and lower level of education. Increased risk for mild ID was found for multiple births, second or later-born children, and children with mothers born outside of California. Children born to Hispanic mothers had increased risk of severe ID, as had children born to Asian mothers who also had decreased risk of mild ID.	*Mild ID:* Male OR 1.90 (1.80–2.00) Low birth weight (LBW) OR 4.90 (4.60–5.20) Black OR 2.10 (1.90–2.30) Maternal age > 35 OR 1.00 (0.90–1.20) High education OR 0.30 (0.30–0.50) Multiple births OR 3.00 (2.70–3.30) Later-born OR 1.60 (1.50–1.70) Asian OR 0.60 (0.50–0.70) *Severe ID:* Hispanic OR 1.40 (1.10–1.80)	*Mild ID:* Male aOR 1.90 (0.80–2.00) LBW aOR 4.30 (4.00–4.60) Black aOR 1.50 (1.40–1.70) Maternal age > 35 aOR 1.40 (1.20–1.60) High education aOR 0.40 (0.30–0.40) Multiple births aOR 1.20 (1.10–1.40) Later-born aOR 1.30 (1.20–1.50) Asian aOR 0.70 (0.60–0.80) *Severe ID:* Hispanic aOR 1.20 (1.00–1.40) Asian aOR 1.30 (1.00–1.70)	High
Heuvelman et al. (68) Sweden	Multivariate regression, *Generalized estimating equations* (GEE) Odds ratios	Sex, parity, maternal age, gestational diabetes (GDM), hyper-tension, pre-eclampsia, parental country of birth, psychiatric history, family income, family size, parental education.	Risk of ID was greatest in those born extremely early, lessening with advancing GA but increasing post term. Risk of ID was greatest among those showing evidence of fetal growth restriction especially before or after term.		Risks of ID At 24 weeks GA aOR 14.54 (11.46–18.44]. At 32 weeks GA aOR 3.59 (3.22–4.01]. At 37 weeks GA aOR 1.50 (1.38–1.63) At 39 weeks GA aOR 1.10 (1.04–1.17) At 42 weeks aOR 1.16 (1.08–1.25) At 44 weeks aOR 1.71 (1.34–2.18)	High
Jones et al. (65) California, United States	Multivariate logistic regression. Odds ratios	Maternal age, ethnicity, birth country, weight, sex, birth year and month	In comparison to general population, higher mid-gestational maternal serum levels of GM-CSF, IL-1α, IL-6, and IFN-γ associated with an increased risk of ASD + ID. The principal association in the DD group relative to GP controls was a decrease in risk with elevated levels of IL-8 and MCP-1.	The only significant associations in the ID group relative to GP controls was a decrease in risk with elevated levels of IL-8 and MCP-1.	ID vs. GP (reduced) IL-1B: aOR 0.91 (0.83–1.00) IL-8: aOR 0.81 (0.70–0.93) MCP: aOR 0.76 (0.59–0.98)	High
Langridge et al. (64) Western Australia, Australia	Logistic regression Odds ratios	Birth year, maternal pregnancy, labor and delivery factors, neonatal outcomes, sociodemographics.	Mild-moderate ID was associated with pregnancy hypertension, asthma, urinary tract infection, some types of antepartum hemorrhage (APH), any type of preterm birth, elective cesarean-sections, breech presentation, poor fetal growth and need for resuscitation at birth. Severe ID was associated with poor fetal growth and need for resuscitation, and any labor or delivery complication.		Mild-moderate ID: Asthma aOR 1.28 (1.12–1.47) Urinary tract infections aOR 1.36 (1.19–1.55) Other APH aOR 1.41 (1.19–1.68) Pregnancy hypertension aOR 1.39 (1.25–1.54), Spontaneous/pre-labor rupture of membranes aOR 1.63 (1.42–1.87) Medically indicated Preterm birth aOR 1.25 (1.04–1.49) Elective CS aOR 1.17 (1.03–1.32) Breech presentation aOR 1.33 (1.13–1.56) POBW < 75%, mild ID aOR 2.33 (1.97–2.75) and severe ID aOR 3.77 (2.15–6.61) Resuscitation at birth, mild ID aOR 1.41 (1.31–1.52) and severe ID aOR 2.18 (1.61–2.94). Male sex, mild ID aOR 1.61 (1.50–1.73) and severe ID aOR 1.67 (1.24–2.24).	Med
Leonard et al. (61) Western Australia, Australia	Multivariate logistic regression Odds ratios	Marital status, maternal country of birth, health insurance status, paternal occupation, geographic remoteness, birth year, and SES	Inappropriate intrauterine growth, less than or greater than optimal birth weight, increased risk of ID. Effects were similar among Caucasian and Aboriginal children.		Caucasian: *Mild-moderate ID:* Severe growth restriction preterm (<37 weeks) aOR 1.71 (1.06–2.77). *Severe ID:* Term births aOR 4.79 (2.59–8.83) Growth restriction aOR 3.20 (1.30–7.90) Poor head growth aOR 3.60 (1.40–9.00)	Med
Louhiala (58) Central and Southern Finland	Logistic regression, Multi-variate analyses. Odds ratios	Adjustment was made in multivariate analysis but covariates unknown	Increased risk of any ID with multiparity, low GA, multiple pregnancy, low social class, male sex, hyperbilirubinemia, hypoglycemia, SGA. Smoking, hypertension, preterm birth (PTB), and maternal advanced age did not increase adjusted odds of ID.	Risk of any ID GA < 33 weeks: OR 17.10 (2.30–130.40) 15 min Apgar score < 7: OR 14.40 (1.80–113.80) Maternal epilepsy: OR 12.00 (0.70–217.60) 5 min Apgar score < 7: OR 7.90 (2.70–22.90) Hypoglycemia: OR 6.50 (2.70–15.60) Multiple birth: OR 6.00 (1.70–20.60) BW < 1500 g: OR 5.60 (1.20–25.70) SGA: OR 4.50 (2.60–8.90) BW < 2500 g: OR 3.40 (1.90–6.10) GA < 35 weeks: OR 2.80 (1.30–5.80) Hyperbilirubinemia: OR 2.70 (1.20–4.10) Breech presentation: OR 2.70 (1.20–4.10) Male sex: OR 1.60 (1.20–2.10) Lowest social class: OR 1.40 (1.90–1.90) Multiparity (>2): OR 1.30 (1.00–1.80)	Risk of any ID GA < 35 weeks: aOR 0.50 (0.20–1.40) Hypoglycemia: aOR 3.60 (1.40–3.70) 1-min Apgar score < 7: aOR 4.80 (2.30–10.10) SGA: aOR 4.40 (2.10–9.30) Multiple birth: aOR 5.60 (1.40–22.30) Hyperbilirubinemia: aOR 3.00 (1.20–7.60) Male sex: aOR 1.50 (1.10–2.10) Lowest social class: aOR 1.70 (1.20–2.40) Multiparity (<2): aOR 1.50 (1.00–2.10)	Low
Luu et al. (57) Providence, Portland and New Haven United States	Linear, logistic regression. Between group means	Sex, maternal age, maternal education, minority status, household structure.	Preterm children obtained scores 6-14 points lower than term controls on all psychometric tests. Severe neonatal brain injury was the strongest predictor of poor intelligence. Antenatal steroids, higher maternal education, and 2-parent family were associated with better cognition.		IQ adj. mean diff for all children preterm vs. term: -13.70 (-17.10, -10.30) (*p* < 0.005) IQ adj. mean diff preterm vs. term excluding brain injury children: -11.70 (-14.70, -8.70) (*p* < 0.005)	Low
McDermott et al. (50) California, United States	Multiple logistic regression Risk ratios	Maternal age, race, education, smoking, alcohol use, pregnancy conditions, congenital infant anomalies, resuscitation at birth.	Strong association between LBW and mild ID for both Blacks and non-Blacks at age 5. Association only held for Blacks in the cohort of children aged 9–11.		Risk of ID in LBW (<2500 g) infants at age 5 years: All aOR 3.40 (2.10–5.40) Black aOR 2.70 (1.40–5.40) Non-Black aOR 3.90 (2.00–7.50) Risk of ID in LBW (<2500 g) infants at age 9–11 years All aOR 1.20 (0.70–2.00) Black aOR 4.10 (1.40–12.00) Non-Black aOR 0.80 (0.40–1.60)	Med
Mervis et al. (47) Atlanta, United States	Logistic regression Odds ratios	Child sex, birth order, GA, maternal age, race, education	Risk of ID was increased for LBW (<2500 g) infants, higher for very low birth weight (VLBW) (<1500 g) and for severe ID. Preterm infants with normal birthweight also had increased risk.		Risk of mild ID: <2500 g aOR 2.40 (1.60–3.80) <1500 aOR 11.70 (2.50–54.70) Risk of severe ID: <2500 g aOR 4.40 (2.60–7.50) <1500 g aOR 29.40 (5.80–148.20)	High
Schieve et al. (40) United States	Multivariate logistic regression Relative risks and standardized morbidity ratios	Child sex, birth order, maternal race-ethnicity, education, age, marital status, and smoking during pregnancy	Children with any ID had higher rates of PTB, LBW, SGA, and low Apgar score than expected based on the US birth cohort, with a higher percentage of mothers who smoked during pregnancy.		Relative risks for any ID PTB SMR aRR 2.10 (1.90–2.20) VPTB aRR 5.40 (4.80–5.90) LBW aRR 3.20 (3.00–3.40) VLBW aRR 7.80 (7.00–8.70) Term aRR LBW 2.40 (2.10–2.70) SGA aRR 1.90 (1.80–2.00) VSGA aRR 2.30 (2.10–2.50) Term SGA aRR 1.60 (1.50–1.70) Low Apgar aRR 8.50 (7.60–9.50) HBW aRR 0.60 (0.50–0.70) Large for GA aRR 0.60 (0.50–0.70)	High
Van Naarden et al. (35) United States	Observed vs. expected ratios	Maternal age	The proportions of multiple births in children with ID born in three US states in 1994 were moderately higher than expected based on US population data.		Observed/expected multiple birth 1.34 [0.95–1.73]).	High
**Maternal physical health**						
Blotiere et al. (84) France	Number of events, crude event rates and crude incidence rate ratios (IRRs)	Maternal age, medication, co-morbidities, history of mental or behavioral disorders, sex, GA, BW.	Association between maternal use of *valproic* acid (VPA) during pregnancy and the risk of ID in offspring, with a dose-response relationship. No increased risk of any of the neurodevelopmental outcomes for prenatal exposure to any other antiepileptic drugs (AEDs) studied.		Compared with prenatal exposure to lamotrigine VPA had increased risk of isolated ID aHR = 3.10 (1.50–6.20)	Med
Drews et al. (76) Atlanta, United States	Logistic regression Odds ratios	Sex, maternal age, race, education, SES, parity, and alcohol use.	Smoking during pregnancy was associated with an increase in the prevalence of ID and the risk increased if at least one pack was smoked a day.	Risk of idiopathic ID with smoking at any time during pregnancy: OR 1.74 (1.21–2.50) Risk of idiopathic ID with smoking during 2nd trimester: OR 1.75 (1.20–2.55)	Smoking at any time during pregnancy: aOR 1.44 (0.92–2.27) Smoking during 2nd trimester: aOR 1.53 (0.80–2.46)	High
Griffith et al. (71) South Carolina, United States	Multiple logistic regression Odds ratios	Maternal age, race, education, birth year and sex	Pre-eclampsia was associated with an increase in the odds of ID. LBW was a mediator of the relationship	Risk of any ID in mothers with pre-eclampsia (PET): OR 1.58 (1.33–1.87)	Risk of any ID in mothers with PET: aOR 1.38 (1.16–1.64) Risk of any ID in mothers with PET: aOR 1.28 (1.07–1.52) including LBW in the model	High
Leonard et al. (63) Western Australia, Australia	Logistic regression Odds ratios	Infant sex and birth order, maternal ethnicity, age group, marital status, height, country of birth, health insurance status, paternal occupation. Remoteness, aggregated SES measure.	Increased risk for mild to moderate ID in children whose mothers had renal or urinary conditions, asthma, and diabetes (ORs, 1.23–1.65) and a threefold increased risk for women with epilepsy. Fivefold risk for having a child with severe for mothers with anemia.	Asthma in mothers: mild to moderate ID (OR, 1.52; CI, 1.26–1.83). Diabetes: mild to moderate ID (OR, 1.69; CI, 1.26–2.27). Undiagnosed cardiac murmurs: (mild to moderate ID, OR, 0.99; CI, 0.61–1.60; severe ID, OR, 2.04; CI, 0.65–6.39) Hypertension: mild to moderate (OR, 0.99; CI, 0.58–1.68) or severe ID (OR, 2.48; CI, 0.79–7.77). Renal or urinary condition mild to moderate ID (OR, 2.09; CI, 1.39–3.14), but not severe ID (OR, 1.01; CI, 0.14–7.19). Epilepsy: mild-moderate ID (OR, 3.53; CI, 2.56–4.84). Anemia: severe ID (OR, 5.26; CI, 2.16–12.80).	Stepwise adjusted logistic regression model with mild-moderate ID as outcome: maternal epilepsy (OR, 3.01; CI, 2.10–4.33), maternal renal or urinary condition (OR, 1.65; CI, 1.06–2.56), and maternal asthma (OR, 1.25; CI, 1.02–1.54), maternal diabetes (OR, 1.38; CI, 0.99–1.91; *p* = 0.06). For severe ID, maternal anemia: (OR, 4.93; CI, 2.00–12.09).	Med
Li et al. (59) Boston, United States	Cox regression Hazard ratios	Child birth year, sex, maternal age, parity, smoking and PTB.	Obesity, pre-gestational diabetes (PGD), obesity with PGD and obesity with gestational diabetes (GD) were all associated with an increased risk of ID.		Risk of any ID Obesity: aHR 1.64 (1.09–2.45) PGD: aHR 2.26 (1.25–4.09) Obese & PGD: aHR 3.63 (1.73–7.61) Obese & GD: aHR 2.31 (1.00–5.36)	High
Mann et al. (55) South Carolina, United States	Cox proportional hazards Models Hazard ratios	Maternal age, race, education level, alcohol use, smoking, sex.	Trichomoniasis during pregnancy associated with ID. Second trimester trichomoniasis associated with a threefold increase in odds of mild or severe ID in the public school system		Trichomoniasis in pregnancy Risk of ID: aHR 1.28 (1.12–1.46) Risk of severe ID: aHR 1.83 (1.34–2.40)	High
Mann et al. (54) South Carolina, United States	Multiple logistic regression & GEE Odds ratios	Maternal age, race, ethnicity, education, smoking, sexually transmitted disease, hyper-tension, diabetes, epilepsy, GA, BW.	The risk of ID was greater in children of women with pre-pregnancy obesity; greatest for women with morbid obesity for ID of any severity. Gestational weight change (gain or loss) was not associated with odds of ID.		Risk of any ID. Pre-pregnancy morbid obesity: aOR 1.52 (1.30–1.77) Risk of severe ID. Pre-pregnancy morbid obesity: aOR 1.83 (1.31–2.56)	Med
Mann et al. (53) South Carolina, United States	Logistic regression Odds ratios GEE	Maternal race, education, age, smoking, hyper-tension, sex, GA, BW.	Maternal diabetes associated with an increased risk of ID in offspring.		Risk of ID Any diabetes: aOR 1.09 (1.01–1.19) Chronic diabetes: aOR 1.31 (0.83–2.06) GDM: aOR 1.08 (0.98–1.19) Diabetes uncertain timing: aOR 1.12 (0.95–1.31)	Med
McDermott et al. (51) South Carolina United States	Logistic regression Relative risk	Maternal age, race, alcohol use, infant GA.	Mothers experiencing a UTI during pregnancy had a slightly increased risk of a child with ID. The risk was increased if the mother did not receive antibiotic treatment.	Risk of ID following UTI in pregnancy depending on antibiotic treatment adjusted only for GA. Unfilled script vs. No UTI controls: RR 1.31 (1.12–1.54) Unfilled script vs. filled script for UTI: RR 1.22 (1.02–1.46)	Risk of ID following UTI in pregnancy depending on antibiotic treatment with full adjustment. Unfilled script vs. No UTI controls: aRR 1.31 (1.12–1.54) Unfilled script vs filled script for UTI: aRR 1.22 (1.02–1.47)	Med
McDermott et al. (49) South Carolina United States	Logistic regression and survival analysis Relative risk	Maternal education, race. Infant sex, GA, BW.	Risk of child ID was increased in women with a urinary tract infection (UTI) during pregnancy		Risk of ID following UTI in pregnancy Overall: aRR 1.16 (1.00–1.29) Trimester 3: aRR 1.40 (1.01–1.95)	High
Salonen and Heinonen (41) Finland	Logistic regression Risk ratios	Maternal age, parity, smoking, mode of delivery, birth defects in siblings.	A strong association was found between pregnancy hypertension and risk of ID in the child	Pregnancy hypertension: risk of any ID, RR 6.00 (1.30–27.00)	Pregnancy hypertension: risk of any ID, aRR 6.10 (1.30–28.90)	Low
Takei et al. (37) England and Wales	Generalized linear model, Poisson regression. Risk ratios	N/A.	Increased death rates from influenza were significantly associated with an increase in births of ID individuals 6 months later.	For every 1,000 female deaths from influenza there was a 17% increase in births of individuals with ID 6 months later. RR 1.17 (3.70–31.70)		Low
Tomson et al. (36) Sweden	Multivariate logistic and Cox models with covariates. Adjusted ORs and HRs	Infant age, sex, GA, BW for GA, parity, maternal education, smoking during pregnancy, cohabitation of parents, parental age, parental psychiatric history.	No evidence for an association between paternal exposure to AEDs during the period of spermatogenesis and increased risk for ID in the offspring. But risk of ID increased in children of fathers with epilepsy, this was not related to the AED medication. High risk of ID amongst mothers with epilepsy, and higher risk for those exposed to AEDs during pregnancy, especially VPA.		All for risk of any ID (mild to severe): Fathers with epilepsy, risk of ID aHR 1.80 (1.20–2.90) vs. without epilepsy. Mothers with epilepsy, risk of ID aHR 2.40 (1.80–3.10) vs. mothers without epilepsy. Mothers with epilepsy who used AED during pregnancy, risk of ID for VPA aHR 9.60 (3.50–26.20), for carbamazepine aHR 4.70 (1.80–12.50), for “other” AED aHR 4.70 (1.40–16.40), for lamotrigine aHR 2.50 (0.90–7.20), vs. unexposed to any AED.	High
**Maternal mental health**						
Di Prinzio et al. (77) Western Australia, Australia	Multiple logistic regression Odds ratios	Sex, birth year, parental age, race, place of birth, birth order, marital status, SES, residential remoteness, father’s psychiatric status.	Risks of ID was increased among children of mothers with severe mental illness compared with children of unaffected mothers.	Risk of ID for children of mothers with any severe mental illness OR 2.70 (2.40–3.00) Risk of ID for children of mothers with schizophrenia OR 3.80 (3.00–4.90) Risk of genetic ID for children of mothers with any severe mental illness OR 2.00 (1.54–2.70) Risk of genetic ID for children of mothers with schizophrenia OR 2.40 (1.20–5.10)	Risk of ID for children of mothers with any severe mental illness aOR 1.70 (1.50–1.90) Risk of ID for children of mothers with schizophrenia aOR 1.70 (1.30–2.30) Risk of genetic ID for children of mothers with any severe mental illness aOR 1.60 (1.20–2.20) Risk of genetic ID for children of mothers with schizophrenia aOR 1.60 (0.70–3.60)	High
Fairthorne et al. (72) Western Australia, Australia	Multinomial logistic regression, binary logistic models for composite case groups. Crude and adjusted ORs.	Maternal age, parity and the index birth year group	Compared to mothers with no previous psychiatric contact, those with any psychiatric contact were more than twice as likely to have a child with ID.		Any previous psychiatric contact (compared to mothers without): ID unknown cause aOR 2.14 (1.90–2.40), ID known cause (not DS) aOR 2.13 (1.60–2.80), ASD with ID aOR 1.77 (1.30–2.40). Schizophrenic disorders: any ID aOR 2.53 (1.40–4.50); ID of unknown cause aOR 2.20 (1.10–4.40). Affective disorders: ASD with ID aOR 2.23 (1.50–3.20), ID unknown cause aOR 1.89 (1.60–2.20); ID known cause (not DS) aOR 1.71 (1.10–2.70).	Med
Morgan et al. (46) Western Australia, Australia	Logistic regression Odds ratios	Child sex, indigenous status and birth order	Children of mothers with schizophrenia, bipolar disorder and unipolar depression were at risk of having offspring with ID certain neonatal complications were independent predictors.	Risk of ID in mothers with Schizophrenia: OR 3.20 (1.80–5.70) Bipolar disorder: OR 3.10 (1.90–4.90) Unipolar depression: OR 2.90 (1.80–4.70)	Risk of ID in mothers with: Schizophrenia: aOR 2.20 (1.20–4.30) Bipolar disorder: aOR 2.60 (1.50–4.40) Unipolar depression: aOR 2.70 (1.60–4.50)	Low
O’Leary et al. (45) Western Australia, Australia	Logistic regression GEE Odds ratios Population attributable fractions	Maternal age and birth year, marital status, parity, maternal mental health, maternal illicit drug use, remoteness, SES.	There was a threefold increase in the adjusted odds of ID in children of mothers with an alcohol-related diagnosis recorded during pregnancy. At least 3.8% of cases of ID could be avoided by preventing maternal alcohol use disorder.	Risk of any ID: Non-Aboriginal: Any alcohol diagnosis OR 1.81 (1.53–2.14); During pregnancy OR 3.52 (1.96–6.34) Aboriginal: Any alcohol diagnosis: OR 1.66 (14.2–1.96); During pregnancy: OR 3.12 (2.13–4.56)	Risk of any ID: Non-Aboriginal: Any alcohol diagnosis: aOR 1.44 (1.18–1.75); During pregnancy: aOR 2.89 (1.62–5.18) Aboriginal: Any alcohol diagnosis: aOR 1.66 (14.2–1.96); During pregnancy: aOR 3.12 (2.13–4.56)	Med
Wang et al. (34) South Carolina, United States	Logistic regression GEE models Odds ratios	BW, birth year, maternal age, race, education, BMI, smoking, alcohol use, drug use, nutrition program participation during pregnancy, maternal ID, asthma, anemia, hyper-tension, myalgia, genito-urinary infection, APH, chorioamnionitis, early or threatened labor.	Women with bipolar disorder or major depression had increased odds of child having ID. Male children higher for bipolar, female child higher for major depression.		Mother had major depression: For males aOR 1.34 (1.14–1.59) PAR 2.17% For females aOR 1.59 (1.30–1.95) PAR 4.70%, Maternal bipolar disorder: For males aOR 1.95 (1.53–2.48) PAR 2.85% For females aOR 1.63 (1.20–2.22) PAR 2.05%	Low
**Environmental**						
Emerson et al. (73) United Kingdom	Non-parametric tests; exposure/prevalence rate ratios.	Complex samples module and sample weights to adjust for the initial sampling design and biases in recruitment and retention at each wave.	British children with ID are more likely than their peers to live in localities with high rates of outdoor air pollution. Averaging across ages, children with IDs were 30–33% more likely to be exposed to high rates of diesel particulate matter, nitrogen dioxide, and carbon monoxide, and 17% more likely to be exposed to high rates of sulfur dioxide.	Exposure rate ratios: 9 months (ID = 552, other = 18,000) PM10: 1.31 (1.14–1.50) NO2: 1.25 (1.08–1.44) SO2: 1.15 (0.93–1.39) CO: 1.23 (1.07–1.41) 3 years (ID = 525, other = 14,371) PM10: 1.35 (1.16–1.56) NO2: 1.31 (1.13–1.51) SO2: 1.16 (0.94–1.43) CO: 1.33 (1.16–1.53) 5 years (ID = 528, other = 14,384) PM10: 1.38 (1.20–1.59) NO2: 1.33 (1.15–1.53) SO2: 1.18 (0.98–1.43) CO: 1.37 (1.19–1.58)		Low
Mackay et al. (56) Scotland	Binary logistic regression. Odds ratios	Maternal age, SES, parity, PET, previous spontaneous abortion, GA, sex and gestation-specific BW percentiles, mode of delivery	Rates of ID highest among children conceived in the first quarter of the year (January–March) and lowest among those conceived in the third (July–September).	Association between calendar year quarter of conception and likelihood of ID (referent 3rd quarter) Quarter 1: OR 1.23 (1.18–1.29) Quarter 2: OR 1.18 (1.13–1.23) Quarter 4: OR 1.13 (1.09–1.18)	Association between calendar year quarter of conception and likelihood of ID (referent 3rd quarter) Quarter 1: aOR 1.23 (1.18–1.29) Quarter 2: aOR 1.17 (1.12–1.22) Quarter 4: aOR 1.13 (1.09–1.18)	High
McDermott et al. (48) South Carolina, United States	Logistic regression Generalized additive model Odds ratios	Sex, GA, BW, SGA, maternal age, race, parity, smoking, alcohol use.	The probability of ID increased for increasing concentrations of arsenic (As) and lead (Pb) in the soil.		Increased risk of ID for I unit in change in As soil concentration aOR 1.13 (1.05–1.22) Appropriate for GA aOR 1.15 (1.06–1.25) Increased risk of ID for I unit in change in Pb oil concentration aOR 1.002 (1.000–1.004) Appropriate for GA aOR 1.002 (1.002–1.004)	Med
McDermott et al. (52) South Carolina, United States	Logistical models. Generalized additive models Odds ratios	Sex, GA, BW, SGA, maternal age, race, parity, smoking, alcohol use.	Positive association between increased soil mercury (Hg) and mild ID; and between soil As, barium, and lead levels and severe ID. Hg and As associated with all levels of ID. In the nine case areas of research, ID prevalence was 5.9%. In the control area the prevalence was 3.2%.	Crude odds ratios Arsenic: Mild ID vs. no ID: 1.00 (0.95–1.05) Severe ID vs. no ID: 1.07 (1.02–1.12) Any ID vs. no ID: 1.03 (1.00–1.07) Mercury: Mild ID vs. no ID: 1.71 (1.34–2.19) Severe ID vs. no ID: 0.91 (0.61–1.35) Any ID vs. no ID: 1.35 (1.09–1.67)	Mild ID (*n* = 104) vs. no ID (*n* = 8229): te(Hg) est df = 2.224, chi-square = 11.176, *p* = 0.007. Severe ID (*n* = 258) vs. no ID (*n* = 8229): te(As, Pb) (interaction term) est df = 7.018, chi square = 12.996, *p* = 0.037. Any ID (n = 514) vs no ID (*n* = 8229): te(As,Pb) (interaction term) est df = 1.368, chi square = 6.073, *p* = 0.006 te(Hg) est df = 1.641, chi square = 9.964, *p* = 0.006	High
Onicescu et al. (44) South Carolina, United States	Spatial importance parameter hierarchical logistic regression modeling. Beta weights and parameters	(1) sex, GA maternal age, race, parity, smoking, age of housing, population density, (2) sex, GA, maternal age, race, and parity	Association between ID and arsenic and mercury concentration in soil during pregnancy, controlling for infant sex, GA, maternal age and race.		Beta weights and parameters: Arsenic: β1 (95% CI): 0.013 (0.00074, 0.038) Mercury: β1 (95% CI): 0.11 (0.021, 0.39)	Med
**Genetic or biological**						
Guo et al. (70) Western China	Deviations from Hardy-Weinberg equilibrium, differences in allele and genotype distribution. Odds ratios	None.	Single marker analysis showed a positive association of ID with rs225012 and rs225010. Particularly with rs225012, TT genotype frequency was significantly higher in ID cases than in controls.	TT genotype frequency (x^2^ = 9.18, *p* = 0.00246). Combination of rs225012 and rs225010 (x^2^ = 15.04, df 2, global *p* = 0.000549) rs225012 OR 1.491 (1.033–2.152) rs225010 OR 1.43 (1.006–2.031)		High
Hurtado et al. (67) Florida, United States	Logistic regression Odds ratios	BW, maternal education, sex, race-ethnicity, maternal age, child’s age at entry to WIC program	Mild or moderate ID associated with anemia.		The effect of hemoglobin was significant for risk of mild-moderate ID: aOR 1.28 (1.05, 1.60). Mild-moderate ID risk: low education (aOR 11.94), normal education (aOR 8.32), VLBW (aOR 4.58), LBW (aOR 2.50).	High
Jelliffe-Pawlowski et al. (66) California, United States	Poisson regression. Prevalence ratios (PR)	Sex, race-ethnicity, plurality, GA, BW, maternal age, education, birthplace, and parity.	Children with chromosomal and other structural birth defects at increased risk of ID by age 7 years. Children with non-chromosomal defects were at substantially increased risk for all levels of ID.	Risk of ID, chromosomal and birth defects: PR 26.80 (22.70–31.70). Down syndrome PR: 211.70 (171.30–261.50) Sex chromosome defects: PR 57.40 (23.70–138.60). Non-chromosomal PR: 11.10 (8.70–14.20)	Risk of ID, Down syndrome aPR 178.40 (141.00–225.70) Non-chromosomal aPR 8.90 (6.80–11.50)	High
Shaw et al. (39) California, United States	Logistic regression OR	Maternal race-ethnicity, and gender	Case and control infants had similar percentages of TT and CT genotypes with some differences by ethnicity.		*Hispanic children:* TT genotype aOR 1.90 (0.70–5.00) CT genotype aOR 2.60 (1.10–5.80) Males with TT genotype aOR 5.40 [1.80–16.30]	Low

*Quality rating: high (90%+), med (75–89%), low (<75%), where percent is calculated on the number of “Y” ratings for each of the JBI rating items per study type rating system. OR, odds ratio; RRR, relative risk ratios; POBW, proportion of optimal birth weight; AGA, appropriate-for-gestational-age; GA, gestational age; SES, socioeconomic status; ASD, autism spectrum disorder; ID, intellectual disability; CP, cerebral palsy; PAR, population attributable risks; BW, birth weight; BMI, body mass index; SGA, small for gestational age; HR, hazard ratio; LBW, low birth weight; GEE, generalized estimating equations; GDM, gestational diabetes; APH, antepartum hemorrhage; VLBW, very low birth weight; VSGA, very small for gestational age; PTB, preterm birth; VPTB, very preterm birth; VPA, valproic acid; AED, antiepileptic drugs; PGD, pre-gestational diabetes; UTI, urinary tract infection; As, arsenic; Pb, lead; Hg, mercury; df, degrees of freedom; PR, prevalence ratios.

**TABLE 3:** Summary of ID risk factors across studies.

Risk factor	Studies	Odds ratio (or equivalent)	Summary
Socioeconomic status (Maternal SES Household disadvantage Local area deprivation)	Camp et al. (83)	For Black children, low SES RR 2.54. For White children, low SES RR 3.36.	Across studies, lower maternal socioeconomic status and relative area deprivation increased risk of ID in offspring. Majority of studies reported mild-moderate ID only; unknown if risk attributable to household disadvantage varies across ID level.
	Drews et al. (75)	Low SES (mild ID): aOR 1.60 (1.00-2.50) (Mild ID, IQ 50–70).	
	Emerson (74)	Lower household SES: moderate ID (aOR = 2.34), severe ID (aOR = 2.42), pervasive multiple ID (aOR = 1.81). Area deprivation codes d1-d9 aORs significant for moderate ID (aOR 3.48 (3.38–3.57) at worst area) and severe ID (aOR 1.45 (1.36–1.55 at worst area).	
	Heikura et al. (69)	For any ID: 1966 cohort: SES IV Level 4 unskilled; aOR 1.70 (0.50–5.80). 1986 cohort: SES IV Level 4 unskilled, aOR 2.20 (1.20–3.90). For mild ID, being a farmer in 1966 cohort aOR 3.50 (1.10–11.40); and in 1986 cohort aOR 3.70 (1.40–9.80). For severe ID (IQ < 50), living in a remote area in 1966 cohort aOR 1.90 (1.00–3.60), level IV unskilled worker for 1986 cohort aOR 3.10 (1.40–6.70).	
	Leonard et al. (60)	Mothers from most socioeconomically disadvantaged area: mild-moderate ID OR 5.61 (2.50–3.91).	
	Leonard et al. (62)	Socio-economic disadvantage: mild-moderate ID, aOR 2.56 (2.27–2.97).	
	Louhiala (58)	Lowest social class at highest risk of any ID, aOR 1.70 (1.20–2.40).	
	Rantakallio (42)	Lower social class (farmers) higher risk: Social class I &II: mild ID 0.4 per 1000, moderate ID 0.4/1000. Social class IV: mild ID 2.5/1000, moderate ID 1.9/1000.	
	Stromme and Magnus (38)	Parents in Social Classes IV to V: increased risk of mild-moderate ID, OR 6.30 (0.70–23.50) for IV; OR 5.00 (1.50–15.60) for V.	
Marital status	Leonard et al. (60)	Single mothers, mild-moderate ID OR 2.18 (1.97–2.42).	Children of single women at higher risk of mild-moderate ID.
	Leonard et al. (62)	For mild-moderate ID, never married or single: OR 2.48 (2.30–2.67); widowed, divorced or separated: OR 2.60 (2.13–3.16).	
Child sex	Croen et al. (13)	Male sex, mild ID aOR 1.90 (0.80–2.00).	Male children at higher risk of mild to severe ID; risk increases in children from minority ethnicity (Black children).
	Drews et al. (75)	Male sex: aOR 1.60 (1.20–2.20) for mild ID (IQ 50–70), aOR 1.70 (1.10–2.50) for severe ID (IQ < 50).	
	Langridge et al. (64)	Male sex for mild ID, aOR 1.61 (1.50–1.73), and severe ID aOR 1.67 (1.24–2.24).	
	Leonard et al. (60)	Male sex, risk mild-moderate: OR 1.55 (1.43–1.68), severe: OR 1.83 (1.38–2.43), ASD OR = 3.97 (2.78–5.69).	
	Louhiala (58)	Male sex: any ID aOR 1.50 (1.10–2.10).	
	Oswald et al. (43)	Male American Indian: any ID (mild-severe) aOR 1.60; Male Black: aOR 4.03; Male Hispanic: any ID aOR 1.35; Female Black: any ID aOR 2.58 (type of ID unspecified).	
	Zheng et al. (30)	Male sex for mild ID aOR 1.23 (1.06–1.44); for severe ID aOR 1.14 (1.00–1.73).	
Child birth order	Croen et al. (13)	Later born, mild ID aOR 1.30 (1.20–1.50).	Later born children consistently at higher risk of mild-moderate ID.
	Drews et al. (75)	Children with two or more older siblings: aOR 1.60 (1.10–2.50) (Mild ID, IQ 50–70).	
	Leonard et al. (62)	Fourth or later born: mild-moderate ID, aOR 2.55 (2.27–2.87).	
	Leonard et al. (60)	Sixth or later born: mild-moderate ID, OR 3.13 (2.50–3.91).	
	Heikura et al. (69)	Multiparity (parity = 4) for 1966 cohort: any ID aOR 1.50 (0.60–3.60); 1986 cohort: any ID aOR 2.40 (1.20–4.90). For mild ID in 1986 cohort, parity = 4 aOR 2.40 (1.20–4.90). For severe ID in 1966 cohort, parity = 4 aOR 1.80 (1.00–3.40).	Multiparity increased odds for any ID; mild ID (later cohort) and severe ID (earlier cohort).
Multiple births	Croen et al. (13)	Multiple births, mild ID aOR 1.20 (1.10–1.40).	Children of multiple births at higher risk of ID.
	Louhiala (58)	Multiple births, risk of any ID aOR 5.60 (1.40–22.30).	
	Van Naarden et al. (35)	Proportions of multiple births in children with ID: Observed/expected 1.34 (0.95–1.73).	
Parental employment and education (Maternal education Maternal employment Maternal income Paternal employment)	Camp et al. (83)	For Black children, low maternal education <9 years, any ID (IQ < 70) RR 2.34. For White children, low maternal education <9 years, ID (IQ < 70) RR 3.00.	Across studies, lower maternal education, typically less than 12 years, conferred highest risk of offspring having ID (mild to severe).
	Chapman et al. (82)	Mothers with < 12 years education vs. some post-secondary education: any ID, RRR 7.00 (6.40–7.70); mild-moderate ID RRR 10.90 (9.60–12.30) and severe ID RRR 3.20 (2.60–3.80). Mothers with high school (education = 12 years): EMH RRR 3.80 (3.40–4.40), TMH 1.70 (1.40–2.00).	
	Decoufle and Boyle (79)	Lower maternal education and race-education interaction. Black mothers with <10 years education risk of ID (IQ < 70) aOR 2.90 (1.60–5.40); vs. for White mothers: aOR 9.10 (3.90–21.30). Maternal education (White and Black mothers combined) <8 years: aOR 6.10 (2.20–16.60) vs. >16 years education aOR 0.20 (0.10–0.50).	
	Decoufle et al. (78)	Children of blue-collar workers in textile products or apparel manufacturing industries, highest risk of ID (mild and severe combined): aOR 10.30 (1.30–81.70).	
	Drews et al. (75)	Maternal education <12 years: aOR 4.10 (2.40-6.90). Maternal education = 12 years aOR 1.80 (1.10-2.90) (Mild ID, IQ 50–70).	
	Leonard et al. (60)	Higher risk for fathers not in the workforce: Mild-moderate ID, OR 5.84 (4.61–7.40), severe ID 3.88 (1.87–8.10).	
	Zheng et al. (30)	Mother illiterate compared to senior high school or above, for mild ID aOR 1.74 (1.21–2.52), for severe ID aOR 2.45 (1.65–3.63).	
Young maternal age	Chapman et al. (82)	Maternal age 15–19 any ID RRR 2.40 (2.30–2.60), for EMH (mild), age 15–19 RRR 2.70 (2.50–3.00).	Children of younger mothers (<20 years) have an increased risk of mild-moderate ID
	Leonard et al. (60)	Maternal age <20 years, mild-moderate ID OR 2.09 (1.82–2.40).	
	Leonard et al. (62)	Maternal age <20 years, mild-moderate ID aOR 1.88 (1.57–2.25).	
Advanced maternal age	Chapman et al. (82)	Maternal age >35 years: any ID, highest RRR maternal age 40-44 = 2.20 (1.60–2.80). For TMH only (IQ 25–54) maternal age 35–39 RRR 2.40 (1.60–3.60) and age >40 RRR 3.50 (1.90–6.50).	Older mothers (>35 years), risk of mild to severe ID.
	Croen et al. (13)	Maternal age >35 years, mild ID aOR 1.40 (1.20–1.60).	
	Drews et al. (75)	Maternal age >30: severe ID aOR 1.80 (1.10-3.10). Maternal age >30 years: any ID with other neurological condition aOR 2.00 (1.20–3.30).	
	Williams and Decoufle (33)	Black race mothers aged 30–34, aOR 3.04 (1.02–9.02) and aged over 35, aOR 3.88 (0.91–16.64).	
	Zheng et al. (30)	Maternal age >34 mild ID aOR 1.47 (1.15–1.88) Maternal age>34: severe ID aOR 1.32 (1.14–2.05).	
Paternal age	Leonard et al. (62)	Paternal age > 39 years, mild-moderate ID aOR 1.59 (1.36–1.86).	Limited evidence to suggest an increased risk. For older fathers.
Parental intellectual disability	Zheng et al. (30)	Mild ID aOR 8.81 (6.45–12.06), severe ID aOR 7.10 (5.09–9.91).	Children of parents with ID at increased risk of ID.
Maternal ethnicity/Race	Abdullahi (29)	Indigenous mothers: ID (IQ < 70), aRRR 1.75 (1.60–1.92); CP with ID, aRRR 1.74 (1.25–2.41). Foreign-born low-income mothers: ASD with ID, aRRR 2.34 (0.96–5.70). Upper-middle-income countries: ASD with ID, aRRR, 1.12 (0.70–1.78).	Indigenous and Black mothers consistently confer higher risk to offspring having ID. Insufficient evidence for other ethnicities. Mothers from minority ethnicity generally confer higher risk to offspring.
	Croen et al. (13)	Black race, mild ID (IQ 50–70) aOR 1.50 (1.40–1.70). Asian, mild ID aOR 0.70 (0.60–0.80) (protective). Hispanic, severe ID aOR 1.20 (1.00–1.40). Asian, severe ID 1.30 (1.00–1.70).	
	Drews et al. (75)	Black race: mild ID (IQ 50–70) aOR 1.80 (1.30–2.60).	
	Emerson (74)	Gypsy/Romany ethnicity: MLD (moderate ID) aOR 2.84 (2.64–3.05). Traveller of Irish Heritage: SLD (severe ID) aOR 1.90 (1.38–2.61), MLD aOR 3.52 (3.20–3.88). Pakistani ethnicity: PMLD (pervasive multiple ID) aOR 2.33 (2.08–2.61).	
	Leonard et al. (60)	Indigenous: mild-moderate ID OR 2.83 (2.52–3.18).	
	Leonard et al. (62)	Indigenous: mild-moderate ID aOR 1.60 (1.41–1.82).	
	Oswald et al. (43)	Any ID by Gender/Ethnicity: Male American Indian, aOR 1.60; Male Black, aOR 4.03; Male Hispanic, aOR 1.35; Female Black, aOR 2.58.	
	Williams and Decoufle (33)	Black race for mothers aged 30–34, co-developmental ID (CNS birth defect, with or without another developmental disability) OR 1.91 (0.89–4.09) and aged >35, OR 4.01 (1.45–11.09).	
	Yeargin-Allsopp et al. (32)	Black race: mild ID, aOR 1.80 (1.30–2.60).	
Preterm birth	Bilder et al. (85)	Gestational age <37 weeks: mild and severe ID combined for inclusive group (all ID), aOR 3.07 (1.98–4.76); combined ID for exclusive group (only those without a known genetic cause of ID), aOR 3.58 (2.15–5.95).	Preterm birth <37 weeks increased risk of ID; preterm birth with low birthweight <2,500 g conferred greatest risk.
	Langridge et al. (64)	Medically induced PTB with mild-moderate ID: aOR 1.25 (1.04–1.49).	
	Luu et al. (57)	FSIQ adj. mean diff for all children preterm vs. term: -13.70 (-17.10, -10.30) (*p* < 0.005). FSIQ adj. mean diff preterm vs. term excluding brain injury children: -11.70 (-14.70, -8.70) (*p* < 0.005).	
	Mervis et al. (47)	<2,500 g preterm mild ID aOR 1.90 (0.90–4.30), severe ID aOR 2.60 (1.00–7.10); >2,500 g preterm mild ID 2.20 (1.30–3.90).	
	Schieve et al. (40)	ID (IQ < 70) without ASD: PTB (<37 weeks) aRR 2.10 (1.90–2.20). VPTB (<32 weeks) aRR 5.40 (4.80–5.90).	
	Heuvelman et al. (68)	24 weeks GA, aOR 14.54 (11.46–18.44]; at 32 weeks, aOR 3.59 (3.22–4.01]; at 37 weeks, aOR 1.50 (1.38–1.63); at 39 weeks, aOR 1.10 (1.04–1.17); at 42 weeks, aOR 1.16 (1.08–1.25); at 44 weeks, aOR 1.71 (1.34–2.18) (67).	
Low birthweight	Bilder et al. (85)	LBW <2,500 g: mild and severe ID, aOR 4.07 (2.34–7.09).	LBW <2,500 g consistently increased risk of mild ID, and in one study severe ID.
	Croen et al. (13)	LBW, mild ID aOR 4.30 (4.00–4.60).	
	McDermott et al. (50)	All mild ID: LBW (<2,500 g) at age 5 years: all aOR 3.40 (2.10–5.40); Black aOR 2.70 (1.40–5.40); non-Black aOR 3.90 (2.00–7.50). LBW (<2,500 g) at age 9–11 years: all aOR 1.20 (0.70–2.00); Black aOR 4.10 (1.40–12.00); non-Black aOR 0.80 (0.40–1.60).	
	Mervis et al. (47)	At any term birth: Risk of mild ID: LBW <2,500 g aOR 2.40 (1.60–3.80), VLBW <1,500 g aOR 11.70 (2.50–54.70). Risk of severe ID: <2,500 g aOR 4.40 (2.60–7.50), <1,500 g aOR 29.40 (5.80–148.20). Fullterm birth: <2,500 g mild ID aOR 2.20 (1.20–4.00), severe ID aOR 3.70 (1.70–7.90).	
	Schieve et al. (40)	ID (IQ<70) LBW (<2,500 g) aRR 3.20 (3.00–3.40); VLBW (<1,500 g) aRR 7.80 (7.00–8.70); Term LBW aRR 2.40 (2.10–2.70).	
Birth weight for gestational age, SGA, birthweight percentiles. Gestational age Intrauterine growth (% optimal birth weight), severe growth restriction	Bilder et al. (85)	SGA: mild and severe ID, aOR 3.43 (2.21–5.33).	SGA conferred greater risk of ID in offspring across studies. Risk of ID was greatest in those born extremely early, lessening with advancing GA but increasing post term. Inappropriate intrauterine growth, less than or greater than optimal birth weight, are associated with development of ID.
	Chen et al. (80)	LBW percentile groups vs. reference group (40th–59th percentiles): 10th–24th percentiles aHR 1.43 (1.22–1.67) and 25th–39th percentiles: aHR 1.28 (1.10–1.50) in population analysis; aHR 1.52 (1.00–2.31) and aHR 1.44 (1.00–2.09) in sibling comparison analysis.	
	Louhiala (58)	SGA: aOR 4.40 (2.10–9.30).	
	Schieve et al. (40)	ID (IQ<70) SGA aRR 1.90 (1.80–2.00); VSGA aRR 2.30 (2.10–2.50); term SGA aRR 1.60 (1.50–1.70).	
	Langridge et al. (64)	POBW of <75%: mild ID aOR 2.33 (1.97–2.75) and severe ID aOR 3.77 (2.15–6.61).	
	Leonard et al. (61)	IUGR in Caucasian children: mild–moderate ID for preterm births (<37 weeks) aOR 1.71 (1.06, 2.77), term births (>37 weeks) aOR = 2.42 (1.88, 3.12). Severe ID aOR 4.79 (2.59, 8.83) for term births. SGR overall, severe ID: aOR 3.20 (1.30– 7.90). Excess intrauterine growth: autism with ID, aOR 2.36 (0.93–6.03).	
Maternal smoking during pregnancy	Drews et al. (76)	Smoking overall: aOR 1.44 (0.92–2.27); during 2nd trimester: aOR 1.53 (0.80–2.46).	Smoking during pregnancy increased risk of ID; risk may be greater in later trimesters.
Maternal body weight or diabetes	Heikura et al. (69)	Pre-pregnancy maternal obesity in second cohort, year 1986 any ID risk aOR 2.80 (1.50–5.30). For mild ID in 1986 cohort, BMI>30 aOR 2.90 (1.30–6.10).	Pre-pregnancy or during pregnancy maternal obesity, and maternal diabetes of any stage conferred higher risk of ID in offspring. Gestational weight gain or loss not associated with greater risk.
	Langridge et al. (64)	Maternal diabetes: mild-moderate ID, aOR 1.27 (1.00–1.61).	
	Leonard et al. (63)	Maternal diabetes: mild-moderate ID, aOR 1.38 (0.99–1.91, *p* = 0.06); autism with ID, aOR 2.95 (1.30–6.73).	
	Li et al. (59)	ID only (no ASD): Obesity: aHR 1.64 (1.09–2.45), pre-gestational diabetes (PGDM): aHR 2.26 (1.25– 4.09), obesity with PGDM: aHR 3.63 (1.73–7.61), and obesity with gestational diabetes (GDM): aHR 2.31 (1.00–5.36). AD+ID: obese GDM, aHR 6.53 (2.45–17.38), obese PGDM 9.73 (4.07–23.27).	
	Mann et al. (54)	Risk of any ID (IQ range not refined): Pre-pregnancy morbid obesity, aOR 1.52 (1.30–1.77). Risk of severe ID: Pre-pregnancy morbid obesity, aOR 1.83 (1.31–2.56).	
	Mann et al. (53)	Risk of any ID (IQ ranged not defined): Any diabetes, aOR 1.09 (1.01–1.19); chronic diabetes, aOR 1.31 (0.83–2.06), GDM, aOR 1.08 (0.98–1.19); diabetes of uncertain timing, aOR 1.12 (0.95–1.31).	
Maternal PET or eclampsia, maternal hypertension	Griffith et al. (71)	Mothers with preeclampsia (PET), risk of any ID (IQ range not defined) aOR 1.38 (1.16–1.64), mothers with PET including child with LBW, aOR 1.28 (1.07–1.52).	Maternal PET or hypertension conferred higher risk of ID in offspring, irrespective of LBW.
	Langridge et al. (64)	Pregnancy hypertension: mild-moderate ID, aOR 1.39 (1.25–1.54).	
	Salonen and Heinonen (41)	Pregnancy hypertension: risk of any ID (IQ range not defined) aRR 6.10 (1.30–28.90).	
Paternal epilepsy	Tomson et al. (36)	Fathers with epilepsy, risk of any ID aHR 1.80 (1.20–2.90) vs. without epilepsy. Fathers with epilepsy and antiepileptic drugs (AED) only during conception, risk of any ID aHR 1.15 (0.49–2.70) vs. non AED. Fathers with epilepsy exposed to AED, risk of ID aHR 1.20 (0.60–2.60) vs. fathers with epilepsy unexposed to AED. Fathers with epilepsy exposed to valproic acid (VPA), risk of ID aHR 1.60 (0.50–5.10), vs. no AED.	No evidence for an association between paternal exposure to AEDs and risk of ID. But risk of ID increased in children of fathers with epilepsy, not related to AED medication.
Maternal epilepsy	Leonard et al. (63)	Maternal epilepsy: mild-moderate ID, aOR 3.01 (2.10–4.33), and autism with ID, aOR 5.80 (2.14–15.74)	Maternal epilepsy and the anti-epileptic drug valproic acid conferred higher risk of ID in offspring.
	Blotiere et al. (84)	AED VPA: increased risk for isolated ID aHR 3.10 (1.50–6.20) vs. lamotrigine. Dose-response relationship with VPA; the highest HR of ID was for the highest mean daily dose [=1,500 mg: HR = 7.30 (3.00–17.70)]. Women treated for epilepsy (*n* < 100) AED VPA: risk of any ID aHR 4.00 (1.90–8.60) vs. lamotrigine.	
	Tomson et al. (36)	Mothers with epilepsy, risk of any ID aHR 2.40 (1.80–3.10) vs. mothers without epilepsy. Mothers with epilepsy and AED exposure, risk of any ID aHR 4.80 (2.10–10.80) vs. unexposed to AED. Mothers with epilepsy who used AED during pregnancy, risk of ID for VPA aHR 9.60 (3.50–26.20), for carbamazepine aHR 4.70 (1.80–12.50), for “other” AED aHR 4.70 (1.40–16.40), for lamotrigine aHR 2.50 (0.90–7.20), vs. unexposed to any AED.	
Maternal asthma	Langridge et al. (64)	Mild-moderate ID: aOR 1.28 (1.12–1.47).	Maternal asthma conferred risk of mild-moderate ID in offspring.
	Leonard et al. (63)	Mild-moderate ID: aOR 1.25 (1.02–1.54).	
Maternal trichomoniasis	Mann et al. (55)	Risk of any ID: aHR 1.28 (1.12–1.46); severe ID: aHR 1.84 (1.35–2.51).	Maternal trichomoniasis associated with slightly increased risk of any ID.
Maternal UTI	Langridge et al. (64)	Mild-moderate ID with urinary tract infection (UTI), aOR 1.36 (1.19–1.55).	Across studies, increased risk of ID with UTI during pregnancy. Risk of ID following UTI in pregnancy may be dependent on antibiotic treatment, and confer a higher risk during the third trimester.
	Leonard et al. (63)	Maternal renal or urinary condition: mild-moderate ID, aOR 1.65 (1.06–2.56).	
	McDermott et al. (51)	Unfilled script vs. no UTI controls, any ID or developmental delay, aRR 1.31 (1.12–1.54). Unfilled script vs. filled script for UTI, any ID or developmental delay aRR 1.22 (1.02–1.47).	
	McDermott et al. (49)	UTI during pregnancy, ID (IQ < 70) aRR 1.16 (1.00–1.29); UTI during Trimester 3, ID (IQ < 70) aRR 1.40 (1.01–1.95).	
Anaemia	Camp et al. (83)	Among Black women, lowest recorded hematocrit <32 mg % among Blacks, ID (IQ < 70) RR 1.35.	Maternal or infant anaemia associated with higher risk of ID in offspring; this may be mediated by gestational age.
	Hurtado et al. (67)	Anaemia increased risk of mild-moderate ID: aOR 1.28 (1.05–1.60).	
	Leonard et al. (63)	Maternal anaemia: severe ID, aOR 4.93 (2.00–12.09).	
Maternal inflammation (Mid-gestational maternal cytokine and chemokine levels. Placental inflammation)	Chen et al. (81)	Placental inflammation: IQ <70 at 7 years old, aOR 1.20 (0.97–1.48), IQ 70–79, aOR 1.03 (0.89–1.18); indirect effects of shorter gestational age.	Maternal inflammation during pregnancy associated with greater risk of mild ID, which may be mediated by gestational age. Maternal serum cytokines IL-8 and MCP-1 may have a protective effect, whereas GM-CSF, IL-1α, IL-6, and IFN-γ were associated with an increased risk of ASD with ID.
	Jones et al. (65)	ID v general population, reduced risk: IL-1B, aOR 0.91 (0.83–1.00); IL-8, aOR 0.81 (0.70–0.93); MCP, aOR 1: 0.76 (0.59–0.98).	
Maternal mental illness	Di Prinzio et al. (77)	Any severe maternal mental illness increased risk of genetic or unknown cause ID, aOR: 1.70 (1.50–1.90). Schizophrenia, aOR 1.70 (1.30–2.30).	Any maternal psychiatric disorder or mental health condition increased risk of ID in offspring; including ASD with ID. Across 4 studies, affective disorders, schizophrenia and any mental illness consistently increased risk.
	Fairthorne et al. (72)	Any previous psychiatric contact: ID of unknown cause aOR 2.14 (1.90–2.40), ID of known cause (not Down syndrome) aOR 2.13 (1.60–2.80), ASD with ID aOR 1.77 (1.30–2.40). Substance abuse disorders: ASD/ID aOR 2.26 (1.10–4.80). Schizophrenia: any ASD or ID aOR 2.66 (1.60–4.40) any ID aOR 2.53 (1.40–4.50); ID of unknown cause aOR 2.20 (1.10–4.40). Affective disorders: ASD with ID aOR 2.23 (1.50–3.20); ID of unknown cause aOR 1.89 (1.60–2.20); ID of known cause (not Down syndrome) aOR 1.71 (1.10–2.70).	
	Morgan et al. (46)	For any ID (including borderline ID of IQ 70–74, including “rare disorders”): Bipolar disorder, aOR 2.60 (1.50–4.40). Unipolar depression, aOR 2.70 (1.60–4.50).	
	Wang et al. (34)	For any ID of unknown cause: Bipolar disorder: males aOR 1.95 (1.53–2.48), females aOR 1.63 (1.20–2.22). Unipolar depression: males aOR 1.34 (1.14–1.59), females aOR 1.59 (1.30–1.95).	
Maternal alcohol use	O’Leary et al. (45)	All for any ID (mild-moderate to severe): Non-Aboriginal: Any alcohol diagnosis: aOR 1.44 (1.18–1.75). During pregnancy: aOR 2.89 (1.62–5.18). Aboriginal: Any alcohol diagnosis: aOR 1.66 (1.42–1.96). During pregnancy: aOR 3.12 (2.13–4.56).	Maternal alcohol consumption increased risk of ID.
Environmental (Geographical remoteness Air pollutants Seasonality Soil concentration)	Emerson et al. (73)	Across 6 age waves (9 months, 3, 5, 7, 11, 14 years), risk of living in areas with top 20% rates of air pollutants: any ID (as defined in study, see Tables 1, 2) exposure RRs = 1.23 (1.00–1.49) for diesel particulate matter, 1.24 (1.02–1.50) for nitrogen dioxide, 1.31 (1.10–1.56) for carbon monoxide and 1.38 (1.11–1.70) for sulphur dioxide.	Environmental factors, including air pollutants, seasonality (month of conception), mineral concentrations in soil during pregnancy, and geographical remoteness all increased risk of ID in offspring.
	Mackay et al. (56)	For any ID (IQ range not defined, qualifying for “Special Education”): First quarter conception (January–March) aOR 1.23 (1.18–1.29), quarter 2 aOR 1.17 (1.12–1.22), quarter 4 aOR 1.13 (1.09–1.18).	
	McDermott et al. (48)	Increased risk of any ID of unknown cause (mild to severe grouped): for I unit in change in arsenic soil concentration: aOR 1.13 (1.05–1.22); for 1 unit change in lead oil concentration: aOR 1.002 (1.000–1.004).	
	McDermott et al. (52)	Mercury, arsenic, barium, lead increased risk of any ID of unknown cause. Mild ID: te(Hg) est df = 2.224, chi-square = 11.176, *p* = 0.007. Severe ID: te(As, Pb) (interaction term) est df = 7.018, chi square = 12.996, *p* = 0.037. Any ID: te(As,Pb) (interaction term) est df = 1.368, chi square = 6.073, *p* = 0.006; te(Hg) est df = 1.641, chi square = 9.964, *p* = 0.006.	
	Onicescu et al. (44)	Mild ID (and/or placement in a public school for educable or mild MR), moderate/severe ID (and/or placement in a public school for trainable (moderate) or profound ID), and unspecified ID, all without known cause. Increased risk overall ID (all severities): Arsenic: β1 = 0.013 (0.00074, 0.038). Mercury: β1 = 0.11 (0.021, 0.39).	
	Zheng et al. (30)	Higher risk for rural areas compared to urban, for mild ID aOR 1.93 (1.55–2.41), for severe ID aOR 1.35 (1.09–1.67).	

There is variability in reporting ID severity. Categories of ID severity are classified according to the International Classification of Diseases, Ninth Revision (ICD-9) as mild (IQ = 50–70), severe (IQ < 50), or unspecified (13). Cases classified using DSM IV recommendations (American Psychiatric Association, 1994) follow “mild” (IQ 50–55 to 69); “moderate” (IQ 35–40 to 40–54); and “severe (including profound)” (IQ < 35 or 40) (59). If a classification is not listed, the study did not specify the level of ID, the IQ range, or used different terminology. ID, intellectual disability; RRR, relative risk ratio; CP, cerebral palsy; ASD, autism spectrum disorder; EMH, educable mentally handicapped (mild ID); TMH, trainable mentally handicapped(moderate to severe ID); OR, odds ratio; RR, risk ratio; SES, socioeconomic status; PTB, preterm birth; VPTB, very preterm birth; LBW, low birth weight; VLBW, very low birth weight; SGA, small for gestational age; VSGA, very small for gestational age; HR, hazard ratio; POBW, proportion of optimal birth weight; IUGR, intrauterine growth retardation; BMI, body mass index; PGDM, pre-gestational diabetes; GDN, gestational diabetes; PET, preeclampsia; AED, antiepileptic drugs; VPA, valproic acid; UTI, urinary tract infection.

## Results

Our initial search yielded a total of 8512 articles after exclusion of duplicates (see PRISMA flow diagram, Figure 1). After screening titles and abstracts, 166 full-text articles were retained and assessed for eligibility. An additional five articles were added from the extended time frame, and a further two articles were added from the reference lists of already included articles. Subsequently, 58 articles were included in the data extraction phase.

### Study selection

#### Description of studies

Among the 58 studies (13, 29–85) included in this systematic review, there were 20 population-based cohort studies (13, 29, 36, 42, 45, 46, 56, 60–64, 66–68, 72, 77, 80, 82, 84), 20 other cohort studies (31, 34, 35, 37, 40, 44, 48–55, 59, 69, 71, 73, 81, 83), 14 case–control studies (32, 33, 39, 41, 47, 57, 58, 65, 70, 75, 76, 78, 79, 85), and four studies with cross-sectional study designs (30, 38, 43, 74) (Table 1). These studies were published from 1984 to 2020 and were predominantly based in North America (*n* = 33), Europe (*n* = 13), and Australia (*n* = 10).

The studies were initially grouped according to the main exposures into six themes (Tables 1, 2): (1) sociodemographic, (2) antenatal and perinatal, (3) maternal physical health, (4) maternal mental health, (5) environmental, (6) genetic or biological studies. For some studies, the exposures under investigation traversed more than one category (see Table 3).

The first group (*n* = 17) encompassed sociodemographic variables which included maternal employment, education, maternal ethnicity/race, marital status, socioeconomic status (including family income), geographical remoteness, country of birth, and age group. Sociodemographic variables pertaining to infants were sex and birth order. The second group (*n* = 14) which was classified as “antenatal and perinatal” included preterm birth, low birthweight, and intrauterine growth restriction but excluded maternal physical or mental health as these were included in separate categories. The third group, “maternal physical health” (*n* = 13), comprised among others smoking, obesity and diabetes (including gestational), pre-eclampsia and hypertension, epilepsy, urinary tract infection, and use of anti-epileptic medication. The fourth group, “maternal mental health” (*n* = 5), was classified as any mental health condition or specific groups, such as affective disorders including bipolar, schizophrenia, and maternal alcohol use disorder. The fifth group, “environmental” (*n* = 5), included air pollution, soil pollution, and seasonality. The sixth group only contained four “genetic or biological” studies.

In the first four categories, the majority were cohort studies (*n* = 34) usually incorporating data linkage (*n* = 30) whereas the comparison group was the population of individuals not affected with ID (Table 1). However, some studies, such as when cases were identified from the Metropolitan Atlanta Developmental Disabilities Study (MADDS) (75, 78, 79), used a case–control design and there was also a small number of cross-sectional studies (*n* = 4) (30, 38, 43, 74). The birth cohorts started as early as 1980 (29) and as late as 2011 (84) and had varying durations of follow-up, while children in the United States National Collaborative Perinatal Project (NCPP) were born from 1959 to 1974 and followed for 8 years (86). Children with ID were identified in a range of ways. In Western Australia (WA), it was through linkage to the WA IDEA (Intellectual Disability Exploring Answers) Database (87), in Atlanta through MADDS (78), in South Carolina through multiple sources including ICD codes in Medicaid billing records, the Department of Education and ID Support services (53). ID was generally defined as IQ < 70 on most recent psychometric test on scales, such as the Wechsler Intelligence Scale for Children (WISC) or Stanford Binet. In a substantial proportion of instances, children with a biomedical cause of ID were excluded, given that the risk factors under study would not be appropriate in these cases.

There was variability in the way ID severity was classified (4), for example, as “any ID,” “mild ID and severe ID,” or “mild-moderate ID and severe ID.” Few studies categorized ID as “mild” (IQ 50–55 to 69); “moderate” (IQ 35–40 to 40–54); or “severe (including profound)” (IQ < 35 or 40 according to DSM IV recommendations (American Psychiatric Association, 1994) (60). ID severity was more likely to be classified according to the International Classification of Diseases, Ninth Revision (ICD-9), as mild (IQ = 50–70), severe (IQ < 50), or unspecified (13). Some studies did not specify the level of ID. The unadjusted and adjusted results along with the relevant covariates are presented according to study in Table 2, and in Table 3 the results are presented according to risk factor given the overlap among studies. Some form of multinomial/multivariate logistic regression was used for analysis in most studies with most results presented as odds ratios although a few used Poisson (13, 37, 65) or Cox regression accounting for age at diagnosis (53, 59).

#### Risk factors

The results from the various categories of risk factors are presented in Table 3.

##### Sociodemographic factors

Using a range of different methods to assess socioeconomic status (SES) in Finland (3) (42, 58, 69), the United States (2) (75, 83), Australia (60), and United Kingdom and Norway (1 each) (38, 74), a clear relationship was identified between low SES and presence of ID in the offspring-mainly mild but also moderate or severe (OR range, 1.60–6.30). The consistency of the findings despite different methods provided moderate evidence of certainty. In Australia, a similar pattern was also seen with the offspring of single compared with partnered mothers [OR 2.18, (95% CI, 1.97–2.42)] (60). In studies from the United States (3) (13, 43, 75), Australia (2) (60, 64), Finland (1) (58), and China (1) (30), the increased risk of mild and severe ID for males was demonstrated (OR range, 1.23–1.90) with high evidence of certainty.

Lower maternal education conferred an increased risk of ID (both mild and severe) in the offspring from studies mostly undertaken over 20 years ago in the United States (75, 79, 82, 83). Despite differences in how maternal education was defined and how ID was categorized, the results were consistent across studies (OR range, 2.34–6.10). The findings were similar in a more recent study from China (30).

Both younger (<20 years) (82) and older (>35 years) (13, 75, 82) maternal age (with moderate certainty of evidence) were examined in United States studies and younger maternal age in Australia (60, 62). Children of younger mothers had an increased risk (RRR/OR range, 1.88–2.70) of ID (62, 82) as did offspring of older mothers (OR range, 1.40–3.88) (13, 33, 75, 82). There have been fewer studies investigating paternal age, but one Australian (62) study identified an increased risk [aOR 1.59 (95% CI, 1.36–1.86)] in offspring of fathers >39 years.

Maternal ethnicity/race was examined in five United States studies (13, 32, 33, 43, 75) and three Australian studies (29, 60, 62). We found higher proportions of children with ID in priority populations, including Black, Hispanic, and Indigenous groups, even after adjustment for SES or maternal education in most studies.

##### Antenatal and perinatal factors

Preterm birth was examined in a number of ways. One study compared the results for all ID with ID exclusive of a genetic cause although the ORs were similar for both groups (85). Another compared mild and severe according to the presence or absence of low birthweight (47) and another according to gestational age (68). Yet another compared IQ levels between those born preterm and those born at term (57). Despite the differing methodologies, the risks for ID overall were increased (OR range, 1.90–3.58) (40, 47, 64, 68, 85) and greater for severe ID (47) and at lower gestations (40, 68) contributing to the certainty of evidence.

Low birthweight was assessed in several of the same studies, and the findings were quite similar to those with preterm birth (OR range, 2.40–4.30) (40, 47, 85). The risk increased the lower the birthweight (40), was higher for severe than mild ID (47, 85) and generally consistent across studies, again providing high certainty of evidence. A number of studies also assessed the impact on ID of fetal growth restriction again by measuring in different ways (40, 58, 61, 64, 80, 85), including the use of the algorithm for percentage of optimal birthweight (POBW) (88). Growth restriction conferred increased risk of ID (OR range, 1.71–4.40) (40, 58, 61, 64, 80, 85).

##### Parental physical health

We only identified one study, published in 1996 and with low certainty of evidence, investigating the relationship between maternal smoking and ID in the offspring and finding a slightly increased risk with confidence limits overlapping unity (76). Six studies considered the impact either of pre-pregnancy obesity or diabetes of any type (including pre-gestational and gestational) and their combinations on the risk of ID in the offspring (53, 54, 59, 63, 64, 69). Pre-pregnancy or pregnancy obesity (54, 69), and maternal diabetes at any stage (53, 63, 64) conferred higher risk of ID in offspring (OR range 1.09–2.9), the latter with low evidence of certainty. However, the risks were not uniform across studies with risks being higher when there was pre-pregnancy morbid obesity (OR range, 1.52–3.63) (54, 59), or when autism was associated with ID (59, 63). Gestational maternal weight gain or loss was not associated with a greater risk (54).

As early as 1984, the first investigation of the relationship with pregnancy hypertension identified an increased risk of ID in the offspring [aRR 6.10, (95%CI, 1.30–28.90)] (41). An increased risk but with a lesser effect size [aOR 1.39, (95% CI, 1.25–1.54)] was found in a much more recent study providing very low evidence of certainty for pregnancy hypertension (64). This was very similar in magnitude to that obtained in another study focusing on pre-eclamptic toxemia (PET) (71).

In a 2006 Australian study, an increased risk of mild-moderate ID in children of mothers with epilepsy was first demonstrated [aOR 3.01, (95% CI, 2.10–4.33)] (63) and replicated again in a recent 2020 study [aHR 2.4, (95%CI, 1.8–3.1)] (36), providing a moderate certainty of evidence. There was no increased risk for the offspring of fathers with epilepsy (36). However, this recent study also focused on the risk posed by specific anti-seizure medications showing that the risk was highest with valproate and carbamazepine. An increased risk with valproate was also reported in another 2020 study (84).

Urinary tract infection during pregnancy was assessed in various ways in different studies, including the use of Medicaid (51) and prescription claims (49) and through midwife-collected data (63). All studies showed consistent but small (OR/RR < 1.65) increased risks of ID in the offspring providing moderate evidence of certainty.

Two studies investigated maternal anemia (63, 83), the United States study providing specific results for the Black population where an increased risk was detected (83). In the Australian study, the increased risk was specifically for severe ID [aOR 4.93, (95% CI, 2.00–12.09)] (63). A third study found an association between early childhood anemia and subsequent mild-to-moderate ID (67). Thus, the heterogeneity reduced the certainty of the evidence. Maternal asthma has only been studied using Australian data with a slight (28%) increased adjusted risk for ID in the offspring (63, 64) and maternal trichomonas during pregnancy in one United States study with a similarly small adjusted risk (55). However, no reason was found for downgrading the certainty of evidence. Two recent studies assessing mid-gestational cytokine and chemokine levels as markers of inflammation were not comparable and thus ineligible for GRADE analysis but provided fairly weak evidence of an increased risk of offspring ID despite some impact on developmental progress (65, 81).

##### Mental health

Five studies investigated the relationship between maternal mental health and risk of ID in the offspring, including four from Australia (45, 46, 72, 77) and one from the United States (34). All were published in the last decade, with most using birth cohort designs and data linkage. The striking findings were the increased risk of ID with any severe maternal mental illness [aOR 1.70, (95% CI, 1.50–1.90)] (77) with moderate evidence of certainty but specifically with schizophrenia [aOR 2.53, (95% CI, 1.40–4.50)] (72) and affective disorders, such as bipolar [aOR 2.60, (95% CI, 1.50–4.40)] (46) and unipolar depression [aOR 2.70, (95% CI, 1.60–4.50)] (46). The United States study identified maternal mental health as a high yield target for the prevention of ID (34). One of the Australian studies identified those women who had had a hospitalization for an alcohol-related disorder had an almost 3-fold increase in the adjusted odds [aOR 3.12, (95% CI, 2.13–4.56)] for their children compared with a matched cohort without such an admission, but the certainty of evidence was considered low (45).

##### Environmental factors

A comparatively smaller number of (30, 44, 48, 52, 56) studies investigated the impact of environmental factors on child ID. Three studies from North Carolina (44, 48, 52) estimated the soil concentrations of various metals in the areas close to where mothers were residing during their pregnancies. Increased concentrations of mercury, arsenic, and lead were most likely to be associated with ID in the children. A Scottish study investigated whether there was any relationship between seasonality of birth and subsequent ID by linking maternity with educational databases relating to 801,592 singleton children and found that rates were highest among children conceived in the first quarter of the year and lowest among those conceived in the third quarter (56). Geographical remoteness has been occasionally studied as a risk factor but may depend on location. In the Chinese study, there was a higher risk for children born in a rural setting (30) but such an association was not found in Australia (60, 62).

#### Quality appraisal and certainty of evidence

A high-quality assessment rating was given to 25 of the 58 studies (43.1%), a medium rating for 22 studies (37.9%), and a low rating for 11 studies (19.0%) using the Joanna Briggs Institute method. One common shortcoming was inadequate approaches toward confounding factors. For the case–control studies, lower scoring studies did not measure the exposures in a standard, valid, and reliable way or had a short exposure time. Cross-sectional studies with low-quality scores typically did not treat confounding factors adequately or did not have valid and reliable outcome measures.

The certainty of evidence was high for the studies investigating male gender, preterm birth, and low birthweight as risk factors for ID, partly because the magnitudes of association were large but also because there was often a dose response. It was moderate for the studies investigating socioeconomic status, race, maternal age and birth order, and maternal conditions, such as PET, epilepsy, asthma, trichomonas infection, urinary tract infection, and mental illness. Evidence for marital status, parental ID, maternal smoking, diabetes, hypertension, and depression as risk factors for ID was of low certainty.

## Discussion

This comprehensive systematic review identified and confirmed a range of risk factors for ID. The greatest numbers of studies fell either into the sociodemographic or the pre- and perinatal domains. Fewer studies related to maternal physical health and fewer still to maternal mental health or environmental factors. We found very few eligible genetic studies given that the genetic risk factors for ID would be rarely studied in a comparison population.

Several of the studies investigating sociodemographic factors had been undertaken over 20 years ago using infrastructures, such as the MADDS (78) and the United States NCPP1 (83). One of the earliest data linkage studies included in this review was that undertaken in Florida in 2002 where birth records were linked to public school records (82). Despite the limitations of that time period, these studies were generally of a high quality and their findings have stood the test of time. Children of teenage (82), socially disadvantaged (75, 83), and multiparous (75) mothers were first identified as being at particular risk almost 20 years ago, findings which have been confirmed more recently in Australian (62) and United Kingdom (74) datasets. Despite other supporting evidence in relation to low maternal education level (82), with a PAR as high as 10%, it is not clear whether any public health approaches have been undertaken to reduce the risk of ID by recognizing and providing the additional support needed for this group of women (both prenatally and post-natally) and their children (72). Minority ethnicities, such as Indigenous, Blacks, and Hispanics, who are already at increased risk of psychosocial stressors, such as discrimination and poverty, are also over-represented among those with less access to education.

Preterm birth and low birthweight were identified as risk factors in a very early case–control study using the MADDS (47) and have continued to be investigated into more recent years (40, 68). A recent Swedish study sourcing data on singletons from the Stockholm Youth Cohort (birth years 1984–2011) demonstrated that the risk of ID increased substantially as birthweight decreased, as it also did when gestational age reduced (68). In contrast to an earlier Australian study which showed that the effect of growth restriction on subsequent ID was greater in term than preterm infants (61), the Stockholm study identified an interaction with growth restriction such that, as might be expected, those who were preterm and growth-restricted, had the highest risk of ID (68). In another recent Swedish study limited to infants born at >36-week gestation, a marginally greater effect size for risk of ID was found in those born at 37/38 weeks compared with 40 weeks (80). Complementing and building on our findings is another Swedish study published in 2022 which confirmed, as we had reported, that the risk of ID increases as gestational age falls, but also showed that the risk also increases again post-term (89). Again similar to our findings, the risk was greater with severe than with mild/moderate ID. Importantly, in all of these studies (40, 47, 61, 68, 80) including this most recent one (89) adjustment had been made for socioeconomic status and/or maternal education (40, 47, 61, 68, 80).

Evidence for the role of maternal physical and mental health as determinants of ID in the offspring has gradually been accumulating over the last two decades. There is a body of evidence building from Australia (63, 64), Finland (69), the United States (53, 54, 59), and now Sweden (90, 91) in relation to maternal obesity, pre-gestational, and gestational diabetes, all showing some level of increased risks. A recently published study focusing on the association of maternal diabetes with a range of neurodevelopmental outcomes has confirmed these relationships with the greatest odds associated with outcomes that included ID (90). Subsequent to this, another study has been published investigating the relationship between gestational weight gain and the risk of ID. This study found that inadequate gestational weight gain increased the risk as did excessive weight gain but only in women who were already obese and had a pre-pregnancy BMI > 25 kg/m^2^ (91). Yet much still remains unknown about the mechanisms underlying these findings and the opportunities for reducing these risks. Some associations with maternal urinary infection have also been demonstrated (49, 51, 63, 64), but there is likely a need for replication and further research given this could be another potentially modifiable factor. Although it is several years since first reported (63), maternal epilepsy and the association with ID and now particularly ASD in the offspring and the potential role of anti-seizure medications (92) is becoming of greater interest (93). The results of the Swedish study included in our review (36) have since been supported by a Danish study (93) with similar results. The risk has been shown to be greatest for women taking valproate during pregnancy but also increased to a lesser extent for those taking carbamazepine, oxcarbazepine, and clonazepam but not lamotrigine (93). This latest study does have implications for appropriate management of epilepsy in pregnancy to reduce the risk of ID through this avenue.

One study by Wang and colleagues in 2015 set out to identify risk factors among children with unknown cause of ID and estimate the PAR associated with these factors (34). This was a retrospective birth cohort study of 123,922 children born between 2004 and 2010 in South Carolina linked to Medicaid billing records and other data. All potential associations with ID were tabulated and the percentages with and without ID were compared. Odds ratios and PARs were calculated. In addition to other known factors, the study found elevated risks for maternal depression and bipolar disease, factors already identified in Australian data (46). Subsequent studies (72, 77) have further elaborated on these findings. What is not understood is the mechanism underlying these findings. There is much recent research investigating relationships between antidepressant medications and ASD (94, 95) but despite the positive associations, a causal link has not been firmly substantiated. Much less is known about such a relationship with ID (96). Although this could be a potential mechanism for our findings, other possibilities could include epigenetic and intergenerational effects, as postulated for ASD (97, 98).

### Strengths and limitations of the reviewed studies

Given the comparatively low prevalence of ID, with just under 2% of children affected (2), the challenges associated with their identification (4), and the lengthy time (40 years) period of the review, studies were generally of medium-to-high quality with consistencies in the findings. In the absence of the existence of infrastructures, such as the WA IDEA Database (87) established especially for that purpose (99), many studies had used creative methods to identify these children in their respective birth cohorts (49, 56, 82) either using ICD codes from Medicaid claims or linking to education or disability services. Exemplifying the rigor of their research, many authors openly acknowledged the shortcomings of their studies, generally only a consequence of the limitations of the available data. For example, the populations for the South Carolina studies were limited to those covered by the Medicaid program which is only available to low-income families. Therefore, the authors explained that their results might not be generalizable to high-income families (53). Despite this, the results were generally consistent with those from WA where population-based data were used (64). Since the majority of studies were located in North America, Scandinavia, and Australia, factors of importance in low- and middle-income countries may not have been adequately represented. Furthermore, we did not identify any studies measuring biomarkers as numbers of cases and controls would likely be too low to meet our eligibility criteria given the prevalence of ID. For example, a study of neuropeptide and neurotrophin levels included only 60 children with ID and 54 control children (100).

In some studies, the type of educational placement was by necessity used as a surrogate for level of ID (67, 74, 82). A significant proportion of studies appropriately excluded ID of biomedical etiology, for example, Down syndrome from the outcome especially in recent studies, one of which specifically stipulated this in the title (68). Most errors associated with exposure misclassification would have resulted in non-differential misclassification and not systematic bias (68). The studies that did not include covariates tended either to be earlier (38, 42, 60, 82) and/or those where data were not available. Most studies reported adjusted odds ratios and some both crude and adjusted (see Table 2).

### Strengths and weaknesses in relation to other studies

The methodology for this review involved a comparison of the presence of potential risk factors in an ID population with their presence in a control population with a sample size of >100 individuals. Thus, in this study, we were not investigating the generally rare but established causes of ID, many of which have a clear genetic etiology. Other than Down syndrome, the commonest known cause with a birth prevalence of ∼1/1,000 live births (101), other much rarer syndromes include Angelman, ARX, Coffin-Lowry syndrome, Cornelia de Lange, Fragile X, PCDH19, Prader–Willi, and Rett and Williams syndromes (102). In this study rather than identifying these individually rare disorders, we were investigating those determinants or risk factors that increase a child’s propensity to having an ID. These factors, which may act individually or in combination, may be modifiable and thus have an important potential to reduce the risk for a child or a population. Improved knowledge about these factors could help in future to identify children at particular risk from an early age, to minimize these risks, and to prioritize such populations for early intervention programs.

In contrast to ASD where over two-thirds of children have comorbid ID (2), reviews on the determinants of ID are comparatively sparse. Although the challenges and opportunities relating to the epidemiology of ID were reviewed in 2002 (4), there have been few subsequent reviews of the topic. In the 2015 study by Wang and colleagues (34), PARs were calculated for many of the factors identified in our current review in descending order of magnitude as follows: parental ID; maternal education; race; genitourinary infection in pregnancy; maternal asthma; maternal hypertension; maternal bipolar disease; maternal depression and low birthweight. The following year, Huang et al. undertook the first systematic review of determinants of ID, but in contrast to ours, theirs was limited to prenatal, perinatal, and neonatal factors and hence only included 17 studies (19). Their findings were consistent with ours for advanced maternal age, being from a priority population [Black race (United States)], low maternal education, parity, maternal smoking, diabetes, hypertension, epilepsy and asthma, male sex, preterm birth, and low birthweight. However, we also found some effects with young maternal age, advanced paternal age, socioeconomic status, fetal growth restriction, maternal urinary tract infection, and various psychiatric conditions. In 2018, Muller and colleagues used a large French longitudinal population-based preterm (<35 weeks) cohort to study the relative contributions of prenatal complications, perinatal characteristics, neonatal morbidities, and socioeconomic conditions to the occurrence of a NDD. In this already preterm population, it was found that, as well as perinatal characteristics, socioeconomic conditions still played a significant role (103). Furthermore, for a similar purpose, the Mannheim cohort of 362 children born between February 1986 and February 1998 was recruited through a two-factorial design which would allocate them into one of nine groups according to their degree of biological (e.g., preterm, low birthweight, or Apgar) or psychosocial (e.g., parental low education, psychiatric disorder, and early parenthood) risk of later developmental disorders (104). When assessed at the age of 4 years, those children at highest risk for both categories were performing most poorly, demonstrating that the risks were additive. Although we could not include this study in our analysis because the presence or absence of ID was not a specific endpoint, the findings concur very much with the interpretations we have made of our own results.

Despite the clearly established relationship between the impact of lead on the developing brain (105–107), environmental factors have generally been somewhat neglected in recent literature reviews on the determinants of ID, possibly because of the new regulations banning the use of leaded petrol (107). One exception is a recent commentary from the United States which considers the combined impact of environmental exposures intertwined with social stressors on risk of NDDs. The authors used a complex system approach based on the structure originally devised by Bronfenbrenner and Evans (108) and thus provided a framework for reshaping public policy (109). Their article elegantly demonstrates the multilevel contributing factors to neurodevelopmental vulnerability and how they may interact. It begins by considering residential locations and the inequities in education, race, income, and immigration status which exist across localities. Then, it examines the factors which either provide beneficial resources or conversely increase stress at the community level, specifically the many environmental hazards and pollutants, evidence for some of which we have seen in this review. Pregnant women living in communities under stress are at greater risk of unhealthy behaviors and likely mental health problems which compound the risk to their unborn child. Moreover, although we only identified one study on this topic which met the criteria for our review, this UK study found that children with ID were more likely than their peers to be living in residential areas with high rates of air pollution (73). However, there has been further recent commentary on the adverse effects of air pollution on neurological development in children and the biological pathways through which these may be mediated (110).

### Meaning of the review

There is a need for improved strategies both to prevent ID and to intervene as early as possible in those infants and young children at most risk. We would concur with the work of Laucht et al. (104) and more recently, Payne-Sturges et al. (109) that there are vulnerable populations where risk factors, we have identified, such as low SES, poor access to education, minority ethnicity, teenage motherhood, mental illness, alcohol abuse, and obesity, are likely to cluster. It will be important to develop strategies to target the modifiable risk factors in these populations preconception. Furthermore, intervention services, leveraging the neuroplasticity of the rapidly developing brain in early childhood, offer the best hope of optimal outcomes and enduring optimization of quality of life for children at risk of ID (111). Yet, their identification is currently contingent on the presentation of behavioral symptoms of developmental delay that typically emerge after 2–3 years of age, and many children are not identified until they reach school age. As a result, timely institution of intervention services is currently suboptimal, and there is a major need for identification of children at risk of ID on the basis of risk factors, prior to the onset of symptoms, or as early as possible in the neurodevelopmental trajectory. A further next step could be to use this information to develop algorithms that can be applied to population-level datasets and in clinical practice to capture as early as possible families and children at greatest risk of ID. At-risk populations could be monitored and interventions implemented where appropriate—preconception, during pregnancy, or after birth—both to reduce the likelihood of ID and to provide optimal opportunities for these vulnerable infants. The ultimate aim was to ensure that these groups of children are identified early to have equitable access to early intervention that is culturally appropriate in line with the concept of proportionate universalism.

## Data availability statement

The original contributions presented in the study are included in the article/Supplementary material, further inquiries can be directed to the corresponding author/s.

## Author contributions

HL, EG, AMa, KW, AF-J, ES, JD, and AMo generated the outline of this review. HL, EG, and AMo wrote the first draft. ES developed the search strategy in conjunction with other authors and undertook the database searches. ES and HL screened the records. EG and HL further assessed the articles for eligibility. EG, HL, VE, AMo, AMa, SW, and BW undertook the JBI quality appraisals. HL and EG performed the GRADE assessments with assistance from JD. BW, HL, and EG undertook the data synthesis. BW prepared the tables. All authors including AW, MS, ML, KV, GA, and KE revised the manuscript and approved the final version.
