# Supplementary material for: A systematic review of the biological, social, and environmental determinants of intellectual disability in children and adolescents
Source: Front Psychiatry. 2022 Aug 25;13:926681. doi: 10.3389/fpsyt.2022.926681 (PMC9453821; doi:10.3389/fpsyt.2022.926681)
Supplement: Supplementary file 2 [file Table_1.docx]

Supplementary Table 1. Study search terminology

| Outcome keywords | | developmental disabil* or developmental disord* or developmental delay* or learning disabil* or learning disorder* or intellectual disability or mental* retard* or mental* handicap* |
| --- | --- | --- |
| AND | | |
| Risk factors | Parental factors | maternal age or paternal age or parental income or socioeconomic* or sociodemog* or parental education or single mother or single parent or parental employment or parental occupation or ethnic* or migrant* or immigra* or indigenous* or cultural* divers* or linguistic* divers* or remote or rural |
|  | OR | |
|  | Mental health factors | maternal psychiatric disord* or paternal psychiatric disord* or maternal mental health or paternal mental health or Maternal psychopathology or paternal psychopathology or parental psychopathology or maternal stress or paternal stress or parental stress or prenatal stress or antenatal stress |
|  | OR | |
|  | Obstetric factors | parity or birth order or preterm or gestation* or biolog* or obstetr* or familial or genetic or prenatal care or antenatal care or high risk pregnancy or complications |
|  | OR | |
|  | Exposures | maternal smoking or maternal cigarette or pregnancy medication or medication during pregnancy or alcohol during pregnancy or prenatal alcohol exposure or environmental chemical* or mercury or lead or traffic pollution or air pollution |
| AND | | |
| Epidemiology keywords | | risk factor or epidemiology or risk or predictor or aetiology or etiology |
