# Supplementary material for: A systematic review of the biological, social, and environmental determinants of intellectual disability in children and adolescents
Source: Front Psychiatry. 2022 Aug 25;13:926681. doi: 10.3389/fpsyt.2022.926681 (PMC9453821; doi:10.3389/fpsyt.2022.926681)
Supplement: Supplementary file 3 [file Table_2.pdf]

Supplementary Table 2: Confidence rating: Grade assessment of body of evidence

			Downgrading factors	Upgrading factors									
**Outcomes (severity of ID)**	**Number of studies**	**Initial rate of confidence**	**Risk of bias**	**Inconsistency**	**Indirectness**	**Imprecision**	**Publication bias**	**Large magnitude of association**	**Dose response**	**Residual confounding**	**Consistency**	**Certainty of the evidence (GRADE)**	**References**
Male sex (mild,severe,any)	6	Moderate	Not likely	Not likely	Not likely	Not likely	Not likely	No upgrade	N/A	No upgrade	Upgrade	⊕⊕⊕⊕	Croen (2001), Louhiala (1995), Drews(1995), Langridge (2013), Oswald (2001), Zheng (2012)
Preterm birth (any)	3	Moderate	Not likely	Not likely	Not likely	Not likely	Not likely	Upgrade	Upgrade	No upgrade	No upgrade	⊕⊕⊕⊕	Bilder (2013), Schieve(2015), Heuvelman(2018).
Low birthweight (mild)	3	Moderate	Not likely	Not likely	Not likely	Not likely	Not likely	Upgrade	Upgrade	No upgrade	No upgrade	⊕⊕⊕⊕	Croen (2001), McDermott (1993), Mervis (1995)
Black race (mild)	3	Moderate	Not likely	Not likely	Not likely	Not likely	Not likely	No upgrade	N/A	No upgrade	No upgrade	⊕⊕⊕○	Croen (2001), Drews(1995), Yeargin-Allsopp (1995)
Maternal education (mild)	4	Moderate	Not likely	Not likely	Not likely	Not likely	Not likely	No upgrade	N/A	No upgrade	No upgrade	⊕⊕⊕○	Camp (1998), Chapman (2002), Decoufle (1995), Drews (1995)
Socioeconomic status (mild,moderate,severe,any)	5	Moderate	Not likely	Not likely	Serious	Not likely	Not likely	No upgrade	N/A	No upgrade	Upgrade	⊕⊕⊕○	Drews (1995), Emerson (2012), Heikura (2008), Leonard(2011), Louhiala (1995)
Maternal age <20 years (mild-moderate)	3	Moderate	Not likely	Serious	Not likely	Not likely	Not likely	Upgrade	N/A	No upgrade	Upgrade	⊕⊕⊕○	Chapman (2002), Leonard (2011), Williams (1999)
Paternal age>39 years (mild-moderate)	1	Moderate	Not likely	Not likely	Not likely	Not likely	Not likely	No upgrade	N/A	No upgrade	No upgrade	⊕⊕⊕○	Leonard (2011)
Small for gestational age (mild,severe,any)	5	Moderate	Not likely	Not likely	Serious	Not likely	Not likely	No upgrade	Upgrade	No upgrade	No upgrade	⊕⊕⊕○	Bilder (2013), Chen (2020), Louhiala (1995), Schieve(2015), Langridge (2013)
Maternal PET (any)	1	Moderate	Not likely	Not likely	Not likely	Not likely	Not likely	No upgrade	N/A	No upgrade	No upgrade	⊕⊕⊕○	Griffith (2011)
Paternal epilepsy (any)	1	Moderate	Not likely	Not likely	Not likely	Not likely	Not likely	No upgrade	N/A	No upgrade	No upgrade	⊕⊕⊕○	Tomson (2020)
Maternal epilepsy (any,mild-moderate)	2	Moderate	Not likely	Not likely	Serious	Not likely	Not likely	Upgrade	N/A	No upgrade	No upgrade	⊕⊕⊕○	Leonard (2006), Tomson (2020)
Maternal asthma (mild-moderate)	1	Moderate	Not likely	Not likely	Not likely	Not likely	Not likely	No upgrade	N/A	No upgrade	No upgrade	⊕⊕⊕○	Langridge (2013)
Maternal trichomonas (any)	1	Moderate	Not likely	Not likely	Not likely	Not likely	Not likely	No upgrade	N/A	No upgrade	No upgrade	⊕⊕⊕○	Mann (2009)
Maternal mental illness (any)	2	Moderate	Not likely	Not likely	Not likely	Not likely	Not likely	No upgrade	N/A	No upgrade	No upgrade	⊕⊕⊕○	Di Prinzio (2018) Fairthorne (2015)
Maternal age >35 years (mild-moderate,severe,any)	4	Moderate	Not likely	Not likely	Serious	Not likely	Not likely	No upgrade	N/A	No upgrade	Upgrade	⊕⊕○○	Drews (1995), Croen (2001), Zheng (2012) and Williams (1999)
Parental intellectual disability (mild,severe)	1	Moderate	Not likely	Not likely	Not likely	Serious	Not likely	No upgrade	N/A	No upgrade	No upgrade	⊕⊕○○	Zheng (2012)
Marital status (mild-moderate)	1	Moderate	Not likely	Not likely	Not likely	Serious	Not likely	No upgrade	N/A	No upgrade	No upgrade	⊕⊕○○	Leonard (2001)
Later birth order (mild)	4	Moderate	Not likely	Not likely	Serious	Not likely	Not likely	No upgrade	N/A	No upgrade	Upgrade	⊕⊕○○	Croen (2001), Drews (1995), Leonard (2011), Heikura (2008)
Maternal smoking (any)	1	moderate	Not likely	Not likely	Not likely	Serious	Not likely	No upgrade	N/A	No upgrade	No upgrade	⊕⊕○○	Drews (1996)
Maternal diabetes (any,mild-moderate)	3	Moderate	Not likely	Not likely	Serious	Not likely	Not likely	No upgrade	N/A	No upgrade	No upgrade	⊕⊕○○	Li (2016), Mann (2013b), Langridge (2013)
Maternal UTI (any, mild-moderate)	3	Moderate	Not likely	Not likely	Serious	Not likely	Not likely	No upgrade	N/A	No upgrade	Upgrade	⊕⊕○○	Langridge (2013), McDermott (2000) and McDermott (2001)
Maternal depressions (any)	3	Moderate	Not likely	Not likely	Serious	Not likely	Not likely	No upgrade	N/A	No upgrade	No upgrade	⊕⊕○○	Fairthorne (2015), Morgan (2012), Wang (2016)
Maternal alcohol use (any)	1	Moderate	Not likely	Not likely	Serious	Not likely	Not likely	No upgrade	N/A	No upgrade	No upgrade	⊕⊕○○	O'Leary (2013)
Pregnancy hypertension (any,mild-moderate)	2	Moderate	Not likely	Serious	Not likely	Serious	Not likely	No upgrade	N/A	No upgrade	No upgrade	⊕○○○	Langridge (2013), Salonen(1984)
Multiple birth (mild,any)	2	Moderate	Not likely	Serious	Serious	Not likely	Not likely	No upgrade	N/A	No upgrade	No upgrade	⊕○○○	Croen (2001), Louhiala (1995)
Maternal anaemia (mild,severe)	2	Moderate	Not likely	Serious	Serious	Not likely	Not likely	No upgrade	N/A	No upgrade	No upgrade	⊕○○○	Hurtado (1999), Leonard (2006)
