# Supplementary material for: A systematic review of the biological, social, and environmental determinants of intellectual disability in children and adolescents
Source: Front Psychiatry. 2022 Aug 25;13:926681. doi: 10.3389/fpsyt.2022.926681 (PMC9453821; doi:10.3389/fpsyt.2022.926681)
Supplement: Supplementary file 4 [file Table_3.docx]

Supplementary Table 3.Exposures, covariates and category of ID by study.
